# Supplementary material for: Shiga toxin-producing Escherichia coli O157:H7 among diarrheic patients and their cattle in Amhara National Regional State, Ethiopia
Source: PLoS One. 2023 Dec 21;18(12):e0295266. doi: 10.1371/journal.pone.0295266 (PMC10734908; doi:10.1371/journal.pone.0295266)
Supplement: S1 File — (DOCX) [file pone.0295266.s001.docx]

**S1 File:** **Supporting information**

**S1 File. Laboratory Standard Operational Procedure (SOP)**

1. **Type and preparation of bacteriological media used for isolation, identification, and antimicrobial susceptibility test for STEC O157:H7**

**A. Tryptone soya broth**

Tryptone soya broth is used to grow aerobic bacteria and helps to transport samples. It was Prepared according to the manufacturer’s instructions. 30 gm of Tryptone soya broth was added in 1000ml demineralized water and were Shaked until completely dissolved. It was then dispensed into a clean bottle, sterilized at 121^o^C for 15 minutes, and stored for subsequent use.

## B. MacConkey (MAC) agar

## MacConkey agar is used to isolate and differentiate non-lactose fermenting and lactose-fermenting gram negative enteric bacteria. It is composed of peptone(20.0gm/l), Lactose(10gm/l). Bile salt No.3 (1.5gm/l), sodium chloride(5.0gm/l), Neutral red(0.03gm/l), crystal violet(0.001gm/l), agar(15.0gm/l). It was Prepared according to manufacturer’s instructions. So, 51.5 grams of MacConkey agar powder was suspended in a liter of distilled water and boiled until completely dissolved. The mixture was then autoclaved at 121^O^C for 15 minutes to sterilize and then cooled at 50^O^C in a water bath. Its PH was adjusted to 7.1. From the mixture 20ml was then dispensed into 10x100ml petri dishes and allowed it to solidify for the next step use. Samples were inoculated onto MacConkey agar and incubated at 37oC for 24 hours. Red or pink colony on MacConkey agar showed E. coli and were further examined by following standard biochemical test (Indole test, TSI, Citrate, Urea, Motility tests).

**C. Cefixime and tellurite Sorbitol MacConkey agar (CT-SMAC)**

Cefixime and tellurite Sorbitol MacConkey (CT-SMAC) Agar (CM0813, Oxoid Ltd., Basingstoke, Hampshire, England) is composed of peptone (20 gm/l), sorbitol (10 gm/l), bile salts No.3 (31.5 gm/l), sodium chloride (5 gm/l), neutral red (0.03 gm/l), crystal violet (0.001 gm/l) and agar (15 gm/l), It was prepared according to the manufacturer’s instruction. 51.5 gm of the powder medium was suspended in one liter of distilled water and boiled until completely dissolved. The mixture was then autoclaved at 121^O^C for 15 minutes and allowed to cool at 50^O^C. One vial cefixime- tellurite antibiotic was added, and Its pH was adjusted to 7.1. Then 20ml of the mixture was poured into a sterile Petri dish and allowed to solidify at room temperature. Finally; the prepared petri dishes were stored in a refrigerator at 4 to 8 ^O^C for subsequent use. Red or pink colony on MacConkey agar which had indole positive, citrate & urea negative, gas production character was inoculated onto CT- SMAC agar and incubated at 37oC for 24 hours. Non sorbitol fermenter colonies of STEC O157:H7 was examined as colorless colonies And sorbitol fermenter STEC O157:H7 was examined as pink colonies.

**D**.  **Mueller-Hinton agar**

Mueller-Hinton agar (MHA) is used in preforming antibiotic susceptibility testing. The medium (CM0337, Oxoid Ltd., Basingstoke, Hants, England) is composed of 300 gm/l beef dehydrated infusion, 17.5 gm/l casein hydrolysate, 1.5 gm/l starch and 17 gm/l agar, and final pH of 7.3. The medium was prepared according to the manufacturer’s instructions. Accordingly; 38 gm of the powdered medium was suspended into 1 liter of distilled water, and then stirred and boiled to dissolve the medium completely. Then, the medium was sterilized by autoclaving at 121^O^C for 15 minutes and cooled to below 45 ^O^C before use. Thereafter, it was poured into sterile Petri dishes. The plates were left at room temperature to solidify then put upside down in the incubator for 24 hours at 37 ^O^C to check for the sterility and to dry the condensed vapor on the plate cover.

Then, Suspension of test organisms were prepared by picking pure colonies with a sterile wire loop suspended in sterile nutrient broth and incubated for 2 hrs. The density of suspensions to be inoculated were determined by comparing with 0.5 McFarland standards. Then, it was inoculated plates by suspension. There after the selected antimicrobials placed on the plates. The plates were left at room temperature for 30 minutes for diffusion then incubated for 24 hours at 37 ^O^C. finally; the zone of growth or inhibition around each disc was measured in millimeters, using a metal caliper, and recorded as sensitive; intermediate and resistance following the method of CLSI 2021.

**Drugs used for antibiotic susceptibility test**

Antimicrobial tests were done for the following commonly prescribed drugs. The used antibiotic disks were amoxicillin/clavulanate (20/10µg), ceftazidime (30µg), ceftriaxone (30µg), cefixime (5µg), cefuroxime (30µg), sulfamethoxazole/trimethoprim (25µg), ciprofloxacin (5µg), norfloxacin (10µg), tetracycline (30µg), gentamycin (10µg), and chloramphenicol (30µg).

**Biochemical and serological test procedure**

Using sterilized inoculation needle 2 pink colony from MacConkey agar were taken and added to 2ml of 0.85% normal saline, mixed and placed in the incubator at 37^O^C for 2 minutes. Then the colony suspension was taken to perform different biochemical tests;

- **Indole test**፡ The test detect the ability of an organism to produce indole from tryptophan present in the medium. The medium contains tryptophan. A sterile loop was used to take colony suspension and inoculated into a test tube which contains 5ml of the tryptophan medium (HiMedia, India). Then, the tubes were incubated at 37^O^C for 24 hours. After incubation period, 0.5 ml of Kovac’s indole reagent (TR008, Titan Biotech Ltd., Rajasthan, India) was added to the inoculated test tubes. The tubes were subjected to gentle shaking and examined for red color in the surface layer within 10 minutes. A red ring on top of the tube indicated indole positive reaction. This is the main character of *E. coli*. In indole test, it can be observed the motility of the organism. If there were turbidity in the media, it indicated motility positive, but if there were growth in line of inoculation, it indicates motility negative (non- motile).
- **Triple Sugar Iron (TSI):** it is used to differentiate among the different groups of *Enterobacteriaceae* based on their ability to ferment glucose, lactose and/or sucrose. It composed of 1% Lactose, 1% Sucrose, 0.1% Glucose, Sodium thiosulfate, and Phenol Red.

A sterile inoculating loop was used to take sample from suspension and stab needle into the butt of the medium (Oxoid, England). Then, the inoculating needle was withdrawn to the slant and streak back and forth up the slant surface. After that, the tube was caped loosely and incubated aerobically for 24 hours at 35 ± 2 °C. After the incubation period, the tube was examined for an acid, gas and hydrogen sulfide production. Thus, the presence of an acid slant-acid butt (yellow/yellow) and cracks, splits or bubbles (gas production) in the medium indicates positive result for *E. coli*.

- **Simon’s Citrate Agar**: An organism that uses citrate breaks down the ammonium salts to ammonia, which creates an alkaline pH and the color of the medium changed into blue. Simmon citrate agar contains sodium citrate (carbon source), ammonium ion (nitrogen source), and pH indicator is bromthymol blue.

A sterile inoculating loop was used to take sample from suspension and stab needle into the butt of the medium (Oxoid, England). Then, the inoculating needle was withdrawn to the slant and streak back and forth up the slant surface. After that, the tube was cap loosely and incubated aerobically for 24 hours at 35 ± 2 °C. After the incubation period, the incubate was observed if it had blue color recorded as positive for *E. coli* however; if was green in color, it was recorded as negative for *E. coli*.

- **Urea test:** is done to determine a bacteria’s ability to hydrolyze urea to make ammonia using the enzyme urease. Urea broth contains a yeast extract, monopotassium phosphate, disodium phosphate, urea, and phenol red indicator.

A sterile inoculating loop was used to take sample from suspension and stab needle into the butt of the medium (Oxoid, England). Then, the inoculating needle was withdrawn to the slant and streak back and forth up the slant surface. After that, the tube was cap loosely and incubated aerobically for 24 hours at 35 ± 2 °C. the result was recorded as positive if there was Pinkish-red color and negative if no color was changed.

***3. E. coli* O157:H7 Latex agglutination test: -** The *E. coli* O157:H7 latex agglutination assay (R24250, Oxoid Ltd., Basingstoke, Hampshire, England) contains latex particles. *E. coli* O157:H7 Latex test includes 3 latex reagents. The particles in each reagent are coated with a different antibody: one against *E. coli* serotype O157, another against *E. coli* serotype H7, and the third with normal rabbit globulin, to serve as the control latex. When test Latex particles are mixed with fresh colonies of O157 and/or H7 strains of *E. coli*, an immunochemical reaction occurs, resulting in agglutination. No agglutination indicates the test isolate is not *E. coli* O157:H7. The Control Latex reagent identifies non-specific agglutination. The test was done with the following procedure.

**Test procedure**

Note: Allow reagents to equilibrate to room temperature before use. Mix latex reagents thoroughly by gentle agitations. Hold the vials vertically and dispense only freefalling drops.

1. Suspected colonies (non-sorbitol fermenting colonies) isolated from Cefixime Tellurite Sorbitol MacConkey (CT-SMAC) agar which were sub cultured on the nutrient agar was used from 24 hours old culture.
2. For each isolate to be tested, one drop of the *E. coli* O157 test latex was dispended into the well of the test slide (reaction card).
3. In similar manner, one drop of *E. coli* control latex was dispensed into a separate well of the test slide.
4. Then a drop of sterile saline solution was dispended into each of the test slide.

5. Using a plastic stick (provided), a portion of the colonies was removed from the nutrient agar plate and emulsified in E. coli O157 test latex and sterile saline water on the slide as well it was spread over two-thirds of the reaction area. Lastly the plastic stick was discarded properly.

6. Once more using a fresh plastic stick, the process was repeated with the remaining colonies and emulsified in *E. coli* control latex on the slide.

7. Thereafter, the slide was rotated using circular motions for up to 1 minute and observe for the presence of precipitation on the *E. coli* O157 test latex, H7 Latex and control latex. If agglutination occurred with the *E. coli* O157 test latex and the control latex was negative. Then, it was recorded as positive for the *E. coli* O157 serogroup. Similarly; it was recorded as positive, if agglutination was observed in test Latex (O157 or H7) accompanied by no agglutination of Control Latex within 1 minute and negative if no agglutination was observed in the test Latex and the Control latex within 1 minute.
