# Supplementary material for: Shiga toxin-producing Escherichia coli O157:H7 among diarrheic patients and their cattle in Amhara National Regional State, Ethiopia
Source: PLoS One. 2023 Dec 21;18(12):e0295266. doi: 10.1371/journal.pone.0295266 (PMC10734908; doi:10.1371/journal.pone.0295266)
Supplement: Attachment — Submitted filename: Manuscript.pdf [file pone.0295266.s006.pdf]

Shiga toxin-producing *Escherichia coli* O157:H7 among diarrheic patients and their cattle in Amhara National Regional State, Ethiopia

Tigist Engda*[1], Belay Tessema[1], Nebiyu Mesifin[2], Anwar Nuru[3], Teshome Belachew[1], Feleke Moges[1]

[1] Department of Medical Microbiology, School of Biomedical and Laboratory Sciences, College of Medicine and Health Sciences, University of Gondar, Gondar, Ethiopia.

[2] Department of Internal Medicine, College of Medicine and Health Sciences, University of Gondar, Gondar, Ethiopia.

[3] College of Veterinary Medicine and Animal Sciences, University of Gondar, Gondar, Ethiopia.

* Corresponding author: Tigist Engda

TE: tigiengda@gmail.com

BT: bt1488@yahoo.com

NM: hakimulig@gmail.com

AN: hamduanwar@yahoo. com

TB: tesh0926@gmail.com

FM: mogesfeleke@gmail.com

Abstract

Background: Shiga toxin-producing *Escherichia coli* O157:H7 (STEC O157:H7) is a zoonotic pathogen that causes diarrhea, hemorrhagic colitis, and hemolytic uremic syndrome worldwide. This study aimed to determine the prevalence, antibiotic susceptibility, and associated risk factors of STEC O157:H7 among diarrheic patients and their cattle.

Methods: A cross-sectional study was conducted among diarrheic patients and their cattle in Amhara National Regional State, Ethiopia from December/2020 to June/2022. A total of 1,149 diarrheic patients and 229 cattle were included in the study. STEC O157:H7 detection was done using culture, latex agglutination test, and Polymerase chain reaction on diarrheic stool samples and recto-anal mucosal swabs of cattle. Antibiotic susceptibility tests were performed using disk diffusion techniques. Factors associated with STEC O157:H7 infection were identified using binary and multivariable logistic regression.

Result: The overall prevalence of STEC O157:H7 in diarrheic patients and their cattle was 11.1% (128/1149) and 14.4% (33/229) respectively. A high percentage of the study subjects were found in under-five children (34.5%). Age less than 5 (AOR: 4.02, 95%CI:1.608-10.058,P=0.003), and greater than 64 years old (AOR:3.36, 95% CI:1.254-8.986, P=0.016), presence of diarrheic patient in the house (AOR:2.11, 95%CI:1.309-3.390, P=0.002), availability of cattle in the house (AOR:2.52, 95%CI:1.261-5.049, P=0.009), and habit of consuming raw foods (AOR:4.35, 95%CI:2.645-7.148, P=0.000) were risk factors. Antibiotic resistance was shown in 109(85.2%), and 31(93.9%) isolates from diarrheic patients and their cattle respectively. The highest levels of antibiotic resistance were found to tetracycline (54.7%, 69.7%) in diarrheic patients and their cattle respectively. Multiple drug resistance was

also observed among 56(43.8%) and 11(33.3%) isolates in diarrheic patients and their cattle respectively.

Conclusion: Our study showed that high prevalence of STEC O157:H7 in diarrheic patients and their cattle. Therefore, continued education should be given to the community on how to care for animals, proper sanitation, and the impact of raw food consumption.

Deleted[Balew Arega]: continues

Introduction

Diarrheal disease is a major public health problem responsible for high morbidity and mortality Worldwide. It is among the leading causes of outpatient visits, hospitalization, and the global year of life lost (YLL) in people of all ages (1). It shares 4% of all deaths and 5% of health losses to disability in the world (2). It is one of the top ten causes of death, ranking ninth globally, sixth in lower-middle-income countries, and second in low-income countries (3). Ethiopia ranks fifth with the highest burden of diarrhea and pneumonia in the world (4). Diarrhea can be caused by a variety of different gastrointestinal (GI) pathogens, including viruses, bacteria, and protozoa (5). Among bacterial pathogens, *Escherichia coli* (*E. coli*) is the most common etiological agent that causes moderate-to-severe diarrhea in low-income countries (6). This group of *E. coli* is said to be Diarrheagenic *E. coli* (DEC) and is a significant contributor to diarrheal disease throughout the world (7). Shiga toxin-producing *E. coli* O157:H7 is one of Diarrheagenic *E. coli* (8).

Shiga toxin-producing *E. coli* O157:H7 is typically a food-borne pathogen causing gastroenteritis and bloody diarrhea, and sometimes it leads to hemolytic uremic syndrome (HUS), thrombotic thrombocytopenic purpura (TTP), end-stage renal disease (ESRD), and even to death (9).

It is noted that for the first time, STEC O157:H7 emerged as a human pathogen in the USA in the early 1980s, during large-scale outbreaks of hemorrhagic colitis and HUS (10). Since then, it has been epidemiologically and clinically important worldwide. Around half of human cases are sporadic, with a seasonal pattern favoring spring and summer. Reports showed that the distribution of STEC O157:H7 differs between regions, potentially influencing the incidence and severity of human disease (11).

Shiga toxin-producing *E. coli* O157:H7 is a zoonotic bacterial pathogen in ruminant animals like cattle. Cattle are the most important reservoir for STEC O157:H7 (12). However; due to the absence of globotriasylceramide-3 (Gb3) vascular receptors for Shiga toxin, its colonization to gastrointestinal tracts of cattle is usually asymptomatic (13), and even it cannot be endocytosed and transported to other organs (14). As a result, it can accumulate in the terminal part of the large intestine and recto-anal junction (15). Moreover; Shiga toxin-producing *E. coli* O157:H7 can also be found in water, soil, meat, fruit, and vegetable products that are contaminated with ruminants' fecal material (16).

Treatment of STEC O157:H7 infection is mainly restricted to supportive care including balancing fluid levels and electrolytes and monitoring the possible development of microangiopathic complications such as HUS (9). Antibiotic therapy is considered to be not beneficial as several antibiotics have been observed to induce the expression and release of Shiga toxins (10).

Even though reports were showing the prevalence of STEC O157:H7 among diarrheic patients in Ethiopia (17, 18, 19, and 20), still there is limited information in different regions of the country, particularly in the Amhara National Regional State. Therefore, this study

aimed to investigate the distribution and antibiotic susceptibility pattern of STEC O157:H7 among diarrheic patients and their cattle in Amhara National Regional State of Ethiopia.

Materials and methods

Study area and period

The study was conducted in Comprehensive Specialized Hospitals in Amhara National Regional States, namely Debre-Markose, Felege-Hiwot, Dessie, Debre-Tabor, and University of Gondar Comprehensive Specialized Hospitals from December 2020 to June 2022. Amhara National Regional State is the second most populous region in the country, Ethiopia. The region has a total population of 20,769,985 of those, 3,492, 000 (16.8%) were urban inhabitants (21).

Study design

A cross-sectional study design was used to determine the prevalence of STEC O157:H7, its associated factors, and antimicrobial susceptibility patterns among diarrheic patients and their cattle.

Population

The source population is all diarrheic patients who had visited the respective Comprehensive Specialized Hospitals for medical services and their cattle. However; These diarrheic patients who are volunteers for the study and fulfill the requirements including giving informed consent and the required amount of stool samples for laboratory analysis and cattle found in the house of STEC O157:H7 positive patients are the study population.

Inclusion and Exclusion criteria

All diarrheic patients who had visited the selective Hospitals for medical services during the study period and cattle found in the house of STEC O157:H7 positive diarrheic patients were

included. However, patients who did not consent to the study, and patients and cattle who were on antibiotics treatment for the last 7 days of data collection, were excluded in the study.

Sample size and Sampling techniques

The sample size for diarrheic patients was calculated using the statistical formula (22),

$$n = \frac{Z^2 x\, p(1-p)}{d^2}$$

where, n=sample size, P=Prevalence in the target population to have STEC O157:H7 (13.9%) (19), Z=Value of standard normal distribution (Z-statistic) at 95% confidence interval (Z=1.96), d= desired absolute precision (margins of error) for estimating a single population proportion (d=2% =0.02), and the final sample size was 1,149.

A simple random sampling technique was used to select the Hospitals. However, the study participants were selected using systematic random sampling techniques. The sample size was proportionally allocated to the selected Hospitals based on the previous case flow. It was calculated by the formula; $ni = \frac{(\text{Ni x n})}{\text{N}}$, Where ni=sample size allocated for a given site, Ni=total number of diarrheic patients visited in a given site in the last 12 months of data collection (Debire-markose=2776, Felege-Hiwot =2576, Debire-Tabor=2489, Dessie=2489, and University of Gondar=3970), N=total number of diarrheic patients attended in the respective Hospitals in the last 12 months (14,300) and n= the sample size of the study (1,149). Based on this formula, 223, 207,200, 200, and 319 diarrheic patients were allocated to Debire-Markose, Felege-Hiwot, Debere-Tabor, Dessie, and the University of Gondar compressive specialized Hospitals respectively.

In the case of cattle, all cattle found in STEC O157:H7-positive patients' houses were included in the study and were found to be 229. Other than the wastage of materials, this increases the chance of getting positive cases.

Data collection tools

Questionnaire

A pretested structured questionnaire was used to collect the necessary data. After obtaining written consent and/or assent from each participant, socio-demographic data, clinical information, and information on the availability of cattle and other possible risk factors of diarrheic patients for STEC O157:H7 were collected. The questionnaire was prepared in English using published studies and translated into the local language (Amharic). Once data were collected, responses to each questionnaire were re-translated into English for analysis and report.

Sample collection and transportation

Diarrheic stool sample

After written informed consent and/or assent was obtained, a structured questionnaire was used and completed by laboratory technicians on a face-to-face interview basis. The participants were also asked and guided by the laboratory technicians to give the stool sample. The sample was collected with a clean, detergent-free disposable screw-capped bottle, and labeled with the patient's code number. On-spot gross examination of stool samples was performed to note the type of diarrhea. Then, about 2gm of each fecal sample was added to culture tubes containing 10ml tryptone soya broth (TSB) for transportation to the laboratory in a cooler box within 2 hrs. of collection (23).

Recto anal mucosal swab

Recto-anal mucosal swab (RAMS) sample is more sensitive than fecal sampling for determining the prevalence of STEC O157:H7 in ruminant animals (24). The Recto-anal mucosal swab was collected before defecation to minimize fecal contamination (25). It was used aseptically from each cattle by inserting a sterile cotton-tipped swab about 2-3cm into the anus. Using a circular motion, the entire surface of the recto-anal mucosa was swabbed. Each swab was placed into a 5ml culture tube containing 2ml TSB for transportation to the laboratory in a cooler box within 2 hours of collection.

Both tryptone soya broth-enriched samples were stored in a cold box with ice packs to transport to the University of Gondar Microbiology Laboratory and the samples were processed immediately after arrival, otherwise, they were stored at -20°C until processed.

Isolation and characterization of Shiga toxin-producing *E. coli* O157:H7 (S file)

The enriched samples were cultured aerobically at 37°C for 24 hours on MacConkey agar (Oxoid, England). After incubation, isolates' characteristics and reactions on agar media were observed and recorded (26). Five to ten suspected colonies (pinkish color appearance) were sub-cultured on separate nutrient agar (Oxoid, England) and confirmed by the biochemical tests.

The Biochemical tests were performed on pink-color colonies to differentiate *E. coli*. The typical biochemical reactions that are considered as *E. coli* are positive for the Indole test, negative on Simon's citrate agar, and urease test, and fermentation of lactose and glucose using Triple Sugar Iron (TSI) with the production of acid and without hydrogen sulfide (H_2S) production (23,27,28).

Deleted[Balew Arega]: which

The identified *E. coli* isolates were sub-cultured on sorbitol MacConkey agar containing cefixime and tellurite (CT-SMAC) (Oxoid, England) media at 37°C for 24hrs to differentiate Shiga toxin-producing *E. coli* from other *E. coli* strains. Sorbitol fermenters (pinkish colonies) were considered as non-O157:H7 *E. coli* strains whereas the non-sorbitol fermenting isolates (colorless colonies) were supposed to be as presumptively confirmed as Shiga toxin-producing *E. coli* O157:H7 strains (29).

Serological test

All non-sorbitol fermenting colonies from the cefixime and tellurite-containing sorbitol-MacConkey agars (CT-SMAC) were serologically confirmed using RIM™ *E. coli* O157:H7 latex test (R24250, Oxoid, Basingstoke, Hants, England)**.** RIM™ *E. coli* O157:H7 latex contains 3 reagents (30). The particles in each reagent are coated with a different antibody: one against STEC serotype O157, another against STEC serotype H7, and the third with normal rabbit globulin to serve as the control latex. A drop of Latex was dispensed into the circle of the reaction card. Using a loop, 10 separate colonies were taken and added into the circle which contain latex reagent. The test latex particles were mixed with fresh colonies of STEC O157:H7, which is positive by CT-SMAC, and in immunochemical reactions, those undergoing agglutination within a minute were registered as positive for STEC O157:H7. The absence of agglutination indicates that the test isolates are not STEC O157:H7. The control Latex reagent identifies non-specific reactions (30).

Polymerase chain reaction (PCR) analysis

Deoxyribonucleic acid (DNA) extraction of the isolate was done by boiling and centrifugation. The isolates grown in nutrient broth were harvested, centrifuged at 12,000 rpm for 10 minutes, and the supernatant was discarded. The sediment was then washed with 1.0ml distilled water

and vortexed. The isolate sediment was lysed by boiling in a water bath at 95^{0}C for 10 minutes and the lysate was then centrifuged. Finally, the supernatant was used as a DNA template for PCR (31).

Amplification of rfb O157 and flic H7 genes

Isolates were confirmed as STEC O157:H7 by using specific primers for rfb O157 (292 bp) (32, 33) and flic H7 genes (625bp) (32, 34). Amplification of rfb O157 and flic H7 are genes encoding for the O157 somatic and H7 flagellar antigens respectively. These were estimated by using adapted primers (33, 34) (Table 1). The amplification mixture contains 5µl platinum super II green master mix (Taq DNA polymerase, dNTPs, $MgCl_{2}$, and reaction buffer at optimal buffer concentration for efficient amplification of DNA templates by PCR, 1.25µl purified DNA, 0.25µl of each forward and reverse primer, then the volume was completed to 3.25µl by deionized water. A negative control was performed by adding 10µl of sterile deionized water and a positive control was performed by adding 1.25µl of known DNA sample (STEC O157:H7; ATCC 43895). All tubes were centrifuged in a microcentrifuge for 10 seconds. Then for the amplification reaction, PCR tubes were transferred to the thermocycler. Polymerase chain reaction conditions consisted of an initial 98°C denaturation step for 1 minute followed sequentially by 35 cycles of 98°C for 10 seconds, 53°C for 10 seconds, and 72°C for 45 seconds. The final extension cycle was followed at 72°C for 5 minutes.

Deleted[Balew Arega]: encoded

Agarose gel electrophoresis

Amplified PCR products were analyzed by gel electrophoresis in 1% agarose gel. The gels were stained with 0.5µl of ethidium bromide (EtBr) per ml and electrophoresed at 120v for 50 minutes using 1xTris-borate-EDTA (TBE) buffer and used a marker DNA ladder of 100bp.

The products were visualized with UV illumination and imaged with a gel documentation system.

Table1. Oligonucleotide sequences of the primers used in PCR amplification (fig1).

Target gene	Primers	Nucleotide sequence (5'-3')	Amplicon size (bp)
rfbO157	rfb-F	GTGTCCATTTATACGGACATCCATG	292
	rfb-R	CCTATAACGTCATGCCAATATTGCC	292
flic H7	flic-F	GCGCTGTCGAGTTCTATCGAGC	625
	flic-R	CAACGGTGACTTTATCGCCATTCC	625

Key: rfb-F=Forward primers, rfb-R=Reverse primers, rfbO157= rfb O157 gene encoding for the O157 somatic antigens, flicH7= flic gene encoding H7 flagellar antigen.

Antimicrobial Susceptibility Test

Antimicrobial susceptibility testing was done on Mueller-Hinton agar (Oxoid, England) using the disk diffusion technique according to the Kirby-Bauer method (18). The antimicrobial agents tested were amoxicillin/clavulanate (20/10μg), ceftazidime (30μg), ceftriaxone (30μg), cefixime (5μg), cefuroxime (30μg), sulfamethoxazole/trimethoprim (25μg), ciprofloxacin (5μg), norfloxacin (10μg), tetracycline (30μg), gentamycin (10μg), and chloramphenicol (30μg).

Bacterial inocula were prepared by suspending 4 to 5 freshly grown STEC O157:H7 colonies in 3-5ml sterile physiological saline and turbidity was adjusted to a 0.5 McFarland standard (18). The sterile cotton swab was dipped, rotated several times, pressed against the wall of the test tube, and then swabbed over the entire surface of the Muller-Hinton agar. After the plates were dried, antibiotic-impregnated disks (Oxoid, England) were placed on the surface of the inoculated plates using sterile thumb forceps. The plates were incubated aerobically at 37°C

for 24 hours. Finally, the diameter of the inhibition zone formed around each disk was measured on the black surface using a transparent ruler placed over the plates. Following CLSI guidelines, the antibiotic susceptibility profile was classified as sensitive (S), intermediate (I), and resistant (R) (35,36,37).

Deleted[Balew Arega]: by

Moreover; Multiple Drug Resistance, resistance to at least three antibiotic classes, profiles were determined against the commonly used classes of antimicrobials, Cephem class (ceftriaxone, ceftazidime, cefixime, cefuroxime), β-lactam combination class (amoxicillin/clavulanate), Aminoglycoside class (gentamycin), Fluoroquinolone class (ciprofloxacin, norfloxacin), Tetracycline class (tetracycline), Folate pathway inhibitors (sulfamethoxazole/trimethoprim), Phenicols class (chloramphenicol) (35).

Study Variables

Dependent variables. STEC O157:H7 associated diarrhea

Independent variables: are explanatory variables that include socio-demographic behavioral and environmental factors concerning diarrheic patients (age, sex, residence, another diarrheic person in the house, duration of diarrhea, frequency of diarrhea, stool appearance, drinking water source, place of defecations, history of hand washing habit, history of consuming raw food, availability of cattle in the house and sharing of home with cattle), and socio-demographic behavioral factors related to cattle found in the diarrheic patient (age, residence, water source, washing habit of cattle, home of cattle, type of cattle food, purpose of cattle).

Quality control

The questionnaire was pretested for a week before data collection started. The training was prepared for laboratory technicians to ensure their data collection techniques. Supervision was made at regular times. The questionnaires were checked their completeness and collected at

Deleted[Balew Arega]: prior to

regular times. Appropriate standard strains, *E. coli* (ATCC 25922), and STEC O157:H7 (ATCC 43895) were used as control strains. The test kits of STEC O157:H7 have their quality control material that can be run in parallel with samples, and all test procedures were done strictly following the manufacturer's instructions.

Data Analysis

Data were checked for completeness and entered and analyzed using IBM SPSS Statistics version 25. Descriptive statistics aimed to summarize the study participants' characteristics across the outcome variable were used. The association between the outcome variable (detection of STEC O157:H7 in diarrheic patients) and each independent variable was analyzed using bi-variable and multi-variable logistic regression models. All the variables were entered into the multivariable logistic regression using the backward LR method to control the confounding effect.

The explanatory variable which had a significant association with the STEC O157:H7 infection in diarrheic patients at a $\leq$ 0.2 in the bi-variable logistic regression model was entered into the multivariable logistic regression model to identify the association of risk factors with STEC O157:H7 infection. The assumption of goodness of the model was checked by the Hosmer-Lemeshow test (p= 0.071). The association between the outcome and the independent variable was calculated by using the adjusted odds ratio at a p-value $\leq$ 0.05 and 95% Confidence Interval.

Deleted[Balew Arega]: lemeshow

Data were summarized using frequency tables. For descriptive statistics, standard deviation (SD), frequencies, and percentages were used. In the case of categorical variables, univariable and multivariate analysis with a 95% confidence interval (CI) was performed to measure their association, and a p-value $\leq$ 0.05 was considered statistically significant.

Ethical statement

We obtained ethical clearance from the ethical review board of the University of Gondar (V/P/RCS/051/69). After clearly explaining the aim of the study, the confidentiality of their information, and their full right to refuse or drop participating in the research, all diarrheic patients or their caregiver signed their consent. Participants' woreda, kebele, specific gote, and phone numbers were registered in the questionnaire. We communicated with STEC O157:H7-positive patients to give information about the disease and to collect cattle samples from those who had cattle in their houses. We tried to reach all cattle found in positive diarrheic patients' houses through the developmental agency workers and signed their consent.

Deleted[Balew Arega]: on

Deleted[Balew Arega]: H7

Results

Socio-demographic characteristics of the diarrheic patients

A total of 1149 diarrheic patients were included in this study. Sample distribution was 441(38.4%) from University of Gondar, 207(18.0%) from Felege-Hiwot, 223(19.4%) from Debre-Markose, 200(17.4%) from Debre-Tabor, and 78(6.8%) from Dessie Comprehensive Specialized Hospitals. Of the total diarrheic patients, 613(53.4%) were male and 396(34.5%) were children under five. A high percentage of the study participants, 719(62.6%) were urban residents (Table 2).

Table 2. Socio-demographic characteristics of diarrheic patients

Socio-demographic characteristics	Category	Frequency N (%)	STEC O157:H7	
			Positive N (%)	Negative N(%)
Age	<5 years	396(34.5)	56(4.8)	340(29.6)
	5-14 years	229(19.9)	24(2.1)	205(17.8)
	15-24 years	170(14.8)	7(0.6)	163(14.2)

	25-64 years	142(12.4)	11(1.0)	131(11.4)
	> 64 years	212(18.4)	30(2.6)	182(15.8)
Sex	Male	613(53.4)	67(5.8)	546(47.6)
	Female	536(46.6)	61(5.3)	475(41.3)
Marital status	Single	311(27.1)	35(3.0)	276(24.0)
	Married	784(68.2)	88(7.7)	696(60.6)
	Divorced	54(4.7)	5(0.4)	49(4.3)
Residence	Urban	719(62.6)	62(5.4)	657(57.2)
	Rural	430(37.4)	66(5.7)	364(31.7)
Educational Status	Illiterate	394(34.3)	54(4.7)	340(29.6)
	Only can read and write	259(22.6)	29(2.5)	230(20.0)
	Primary school completed	252(21.9)	20(1.7)	232(20.2)
	Secondary school completed	123(10.7)	16(1.4)	107(9.3)
	University graduated	121(10.5)	9(0.8)	112(9.8)
Occupation	Farmer	165(14.4)	13(1.1)	152(13.2)
	Housewife	288(25.1)	44(3.8)	244(21.3)
	Merchant	308(26.8)	32(2.8)	276(24.0)
	Student	63(5.5)	10(0.9)	53(4.6)
	Government Employer	170(14.8)	15(1.3)	155(13.5)
	Daily laborer	92(8.0)	9(0.8)	83(7.2)
	Private employer	63(5.5)	5(0.4)	58(5.1)

Monthly income	< 500 birrs	395(34.4)	53(4.6)	342(29.8)
	500-1000 birr	384(33.4)	48(4.2)	336(29.2)
	>1000 birr	370(32.2)	27(2.3)	343(29.9)
Another diarrheic person in the house	No	541(47.1)	42(3.6)	499(43.5)
	Yes	608(52.9)	86(7.5)	522(45.4)

Prevalence of Shiga toxin-producing *Escherichia coli* O157:H7

In diarrheic patients, 1149 diarrheic stool samples were collected with the standard procedure. The collected diarrheic stool samples were bloody, mucoid, and watery with respective numbers of 420(36.6%), 390(33.9%), and 339(29.5%), respectively. Of the total stool samples, 128 stool samples were positive for STEC O157:H7 with rfb O157 and flic H7 genes in PCR with agarose gel Electrophoresis tested (Table1). As a result, the overall prevalence was 11.1% (95% CI: 0.09-0.13).

This study showed that the highest percentage of STEC O157:H7 positive diarrheic patients had bloody diarrhea (5.2%), 1 to 3 days duration of diarrhea (5.1%), 4 to 9 times diarrheal frequency (6.7%), history of hand washing habit in before and after eating (9.1%), and history of consuming raw food (8.3%) (Table 3).

Only a small proportion of the study participants, 187(16.3%) had a total of 561 cattle in their house (Table 3). However, from 187 diarrheic patients, only 53 were positive for STEC O157:H7 and had 229 cattle only. Of these cattle, 123, 47, and 59 were cows, oxen, and calves respectively. Samples from these cattle were collected by following standard protocol (23).

Deleted[Balew Arega]: From

In PCR with agarose gel Electrophoresis tested recto mucosal swab samples of these cattle, 33 (18 cows, 6 oxen, and 9 calves) were positive for STEC O157:H7 with rfb O157 and flic H7

Deleted[Balew Arega]: s

genes (Table_1). As a result, the overall prevalence of STEC O157:H7 in cattle was 14.4% (95%CI:0.39-0.52).

Table 3. Clinical manifestation and behavioral characteristics of diarrheic patients

Clinical manifestation and behavioral characteristics	Category	Frequency N (%)	STEC O157:H7	
			Positive N (%)	Negative N (%)
Duration of diarrhea	1 - 3 days	457(39.8)	58(5.1)	399(34.7)
	4 - 6 days	421(36.6)	43(3.7)	378(32.9)
	7 - 10 days	271(23.6)	27(2.3)	244(21.3)
Frequency of diarrhea	≤ 3	724(63.0)	41(3.6)	683(59.4)
	4 – 9	384(33.4)	77(6.7)	307(26.7)
	> 9	41(3.6)	10(0.9)	31(2.8)
Stool appearance	Watery	339(29.5)	20(1.7)	319(27.8)
	Mucoid	390(33.9)	48(4.2)	342(29.8)
	Bloody	420(36.6)	60(5.2)	360(31.3)
Drinking water source	Tap	708(61.6)	59(5.1)	649(56.5)
	Water well	404(35.2)	64(5.6)	340(29.6)
	Surface	37(3.2)	5(0.4)	32(2.8)
Diarrheic patient	No	456(39.7)	75(6.5)	381(33.2)

water treatment	Filtering	391(34.0)	27(2.3)	364(31.7)
	Aqua tab or Water guard	161(14.0)	19(1.6)	143(12.4)
	Boiling	141(12.3)	8(0.7)	133(11.6)
Place of defecations	Modern toilet	217(18.9)	16(1.4)	201(17.5)
	Traditional toilet	583(50.7)	51(4.4)	532(46.3)
	Open field	160(13.9)	30(2.6)	130(11.3)
	Public toilet	189(16.5)	31(2.7)	158(13.8)
History of hand washing habit	Before eating and after the toilet	249(21.7)	13(1.1)	236(20.5)
	Before preparing food and washing house utilities	168(14.6)	10(0.9)	158(13.8)
	Before and after eating	732(63.7)	105(9.1)	627(54.6)
History of consuming raw food	Yes	604(52.6)	96(8.3)	508(44.2)
	No	545(47.4)	32(2.8)	513(44.6)
Availability of cattle in the house	Yes	187(16.3)	53(4.6)	134(11.)
	No	962(83.7)	74(6.4)	888(77.3)
Sharing of home with cattle	Yes	103(9.0)	33(2.9)	70(6.1)
	No	1046(91.0)	95(8.2)	951(82.8)

331 **Associated risk factors for Shiga toxin-producing *Escherichia coli* O157:H7**

In this study, different variables were considered during the bivariate analysis. These variables were taken as a possible risk factor for STEC O157:H7 infections. Comparatively, a higher prevalence of STEC O157:H7 was found among under 5 children and over 64 years of age. Moreover; patients who were rural residents, had mucoid and bloody diarrhea, had low monthly income, were poor in regular hand washing activities before and after eating, had the habit of consuming raw food, and had cattle were risk factors for STEC O157:H7.

Deleted[Balew Arega]: were

In multivariable analysis, diarrheic patients of under-five children were 4(AOR=4.02, 95%CI; 1.608-10.058, P=0.003) times and old ages greater than 64 years were 3(AOR=3.357, 95% CI: 1.254 - 8.986, P=0.016) times more likely to have STEC O157:H7 associated diarrhea than other age groups (Table 4).

Deleted[Balew Arega]: under

Diarrheic patients who had an experience of consuming raw foods were 4(AOR= 4.35, 95% CI: 2.645-7.148, p=0.000) times more likely to have STEC O157:H7 associated diarrhea than their counterparts. Those diarrheic patients who had cattle in their house were 3(AOR=2.52 95% CI: 1.261 – 5.049, P=0.009) times more likely to have STEC O157:H7 associated diarrhea than patients who did not have (Table 4).

Table 4. Associated risk factors of Shiga toxin-producing *E. coli* O157:H7 among diarrheic patients.

Deleted[Balew Arega]:

Variables	Category	COR (95% CI)	P-value	AOR (95% CI)	P-value
Age	<5 years	3.835(1.710-8.601)	0.001	4.02(1.608-10.058)	0.003
	5 - 14 years	2.726(1.146-6.486)	0.023	2.39(0.905-6.337)	0.079
	15 - 24 years	1.00		1.00	
	25 - 64 years	1.955(0.737-5.185)	0.178	1.63 (0.546-4.867)	0.381

	> 64 years	3.838(1.642-8.975)	0.002	3.36 (1.254-8.986)	0.016
Residence	Urban	1.00		1.00	
	Rural	1.921(1.328-2.780)	0.001		
Monthly income	<500 birr	1.969(1.210-3.204)	0.006	1.55 (0.842-2.846)	0.160
	500 - 1000 birr	1.815(1.106-2.977)	0.018	2.39 (1.285-4.456)	0.006
	>1000 birr	1.00		1.00	
Another diarrheic patient in the house	No	1.00		1.00	
	Yes	1.957(1.327-2.888)	0.001	2.11(1.309-3.390)	0.002
Frequency of diarrhea/ day	≤ 3	1.00		1.00	
	4 – 9	4.178(2.795-6.247)	0.000	4.34 (2.730-6.902)	0.000
	> 9	5.374(2.465-11.715)	0.000	4.23 (1.669-10.724)	0.002
Drinking water source	Tap	1.00			
	Water well	2.071(1.420-3.020)	0.000		
	Surface	1.719(0.645-4.577)	0.278		
Drinking water treatment	No	3.273(1.538-6.964)	0.002		
	Filtering	1.233(0.547-2.762)	0.614		
	Water guard	2.093(0.881-4.973)	0.095		
	Boiling	1.00			
Place of	Modern	1.00			

defecation	Traditional Latrine	1.204(0.671-2.161)	0.533		
	Open field	2.899(1.520-5.529)	0.001		
	Public latrine	2.465(1.302-4.667)	0.006		
History of hand washing habit	Before eating & and after the toilet	1.00		1.00	
	Before preparing food and washing house utilities	1.149(0.492-2.685)	0.748	0.783(0.296-2.073)	0.623
	After eating	3.040(1.676-5.514)	0.000	3.55 (1.766-7.143)	0.000
History of consuming raw food	Yes	3.030(1.994-4.604)	0.000	4.35 (2.645-7.148)	0.000
	No	1.00		1.00	
Availability of cattle in the house	Yes	4.872(3.281-7.234)	0.000	2.52(1.261-5.049)	0.009
	No	1.00		1.00	
Sharing of home with cattle	Yes	4.719(2.966-7.500)	0.000	2.03(0.892-4.614)	0.092
	No	1.00			

Antimicrobial susceptibility profile of Shiga toxin-producing *E. coli* O157:H7

All STEC O157:H7 isolates in diarrheic patients and their cattle were subjected to an antimicrobial susceptibility test with 11 commonly prescribed antimicrobial drugs. Antibiotic

resistances were shown in 109(85.2%) and 31(93.9%) STEC O157:H7 isolates from diarrheic patient and their cattle respectively.

Shiga toxin-producing *E. coli* O157:H7 isolates exhibited high level of antibiotic resistance to tetracycline 70(54.7%), amoxicillin/clavulanate 68(53.1%), and sulfamethoxazole/ trimethoprim 56(43.8%) in diarrheic patients, and tetracycline 23(69.7%), Amoxicillin/ clavulanate, 15(45.4%), Sulfamethoxazole/trimethoprim 16(48.5%) in cattle. However; low level of antibiotic resistance was found in chloramphenicol 12(9.4%), norfloxacin 12(9.4%), and ciprofloxacin 16(12.5%) in diarrheic patients, and chloramphenicol 1(3.03%), norfloxacin 2(6.1%) and ciprofloxacin 4(12.1%) in cattle (Table 5).

Table 5. Antibiotic susceptibility profiles for STEC O157:H7 isolates from diarrheic patients and their cattle.

Antibiotic	STEC O157:H7 from diarrheic Patients (N=128)			STEC O157:H7 from Cattle (N=33)		
	S N (%)	I N (%)	R N (%)	S N (%)	I N (%)	R N (%)
Amoxicillin/ Clavulanate	48(37.5)	12(9.4)	68(53.1)	12(36.4)	6(18.2)	15(45.4)
Ceftazidime	106(82.8)	4(3.1)	18(14.1)	25(75.7)	2(6.1)	6(18.2)
Ceftriaxone	99(77.3)	12(9.4)	17(13.3)	18(54.5)	9(27.3)	6(18.2)
Cefixime	101(78.9)	8(6.3)	19(14.9)	17(51.5)	10(30.3)	6(18.2)
Cefuroxime	92(71.9)	2(1.6)	34(26.5)	22(66.7)	2(6.0)	9(27.3)
Sulfamethoxazole/ Trimethoprim	67(52.3)	5(3.9)	56(43.8)	15(45.4)	2(6.1)	16(48.5)
Ciprofloxacin	103(80.5)	9(7.0)	16(12.5)	24(72.7)	5(15.2)	4(12.1)
Norfloxacin	112(87.5)	4(3.1)	12(9.4)	28(84.9)	3(9.0)	2(6.1)

Tetracycline	51(39.8)	7(5.5)	70(54.7)	9(27.3)	1(3.0)	23(69.7)
Gentamycin	63(49.2)	10(7.8)	55(43)	10(30.3)	17(5.5)	6(18.2)
Chloramphenicol	109(85.1)	7(5.5)	12(9.4)	28(84.9)	4(12.1)	1(3.0)

Key: AMC: Amoxicillin/Clavulanate, CAZ: Ceftazidime, CTR: Ceftriaxone, CFM: Cefixime, CXM: Cefuroxime, SXT:, CIP: Ciprofloxacin, NX: Norfloxacin, TC: Tetracycline, CN: Gentamycin, CHL: Chloramphenicol. MDR = Multi-Drug Resistance, S=Susceptible, I=Intermediate, and R= Resistance

Deleted[Balew Arega]: Multi

In isolates from diarrheic patients, only 11.7% were not resistant to any of the selected antibiotics. However; 43.8% showed Multiple Drug Resistance, resistance to at least three antibiotic classes. From these 3.1% and 0.8% of the isolates were resistant to six and seven antibiotic classes respectively (Table 6).

Deleted[Balew Arega]: s

Table 6. Multiple antibiotic resistance patterns of STEC O157:H7 isolates from diarrheic patients

Antibiotic Disk	Total Isolates N (%)	Antibiogram
Isolates sensitive to all antibiotics (19) *	19(11.7)	R0
TC (5), SXT (6), AMC (3), CXM (3), CFM (1), CN (2)	20(15.6)	R1
AMC & TC (5), SXT & CIP (1), SXT & TC (10), AMC & CN (1), AMC& SXT (3), AMC & CXM (2), CXM & TC (4), CIP & CN (1), TC, CAZ & CTR (1), AMC, CTR & CXM (1) CIP, NX & TC (1), AMC, CTR & CFM (3)	33(25.8)	R2
SXT, TC & CN(1), AMC, SXT, & CIP(1), AMC, SXT & CAF(1), AMC, CXM & TC(2), SXT, TC & CN(2), AMC, CAZ & TC (1), AMC, SXT &	26(20.3)	R3

Deleted[Balew Arega]: -

TC(7), AMC, TC & CHL(2), AMC, CXM & CN(3), AMC, TC & CN(1), AMC, SXT & CN(1), SXT, CIP, NX & TC(1), AMC, CFM, CXM & TC(1), AMC, CAZ & CTR, CFM & CN(1), AMC, CAZ, CTR, CFM & TC(1),		
AMC, CTR, SXT & TC(2), AMC, TC, CN & CHL(1), AMC, SXT, TC & CN(1), AMC, CXM, SXT & TC(1), AMC, CN, CHL, CAZ & CXM(2), AMC, CAZ, CXM, TC & CN(1), AMC, SXT, CIP, NX & TC(1), AMC, CAZ, CTR, CFM, SXT & TC(1), AMC, CAZ, CTR, CFM, TC & CN(1), AMC, CAZ, CFM, CXM, TC & CN(1), AMC, CTR, CFM, CXM, SXT & TC (1), CAZ, CFM, CXM, SXT, CIP, NX & TC(1), AMC, CAZ, CTR, CFM, CXM, SXT & TC(2), AMC, CAZ, CTR, CFM, CXM, SXT, CIP & NX(1)	17(13.3)	R4
AMC, CXM, SXT, TC & CHL (1), AMC, CXM, SXT, TC & CN(1), AMC, SXT, TC, CN & CHL(1), AMC, SXT, CIP, NX, TC & CN(1), AMC, CAZ, CXM, CIP, TC & CHL(1), AMC, CAZ, CXM, SXT, TC & CN(1), AMC, CAZ, CTR, CFM, SXT, CIP, NX & TC(1), AMC, CAZ, CTR, CFM, CXM, SXT, CIP, NX & TC(1)	8(6.3)	R5
AMC, CXM, SXT, TC, CN & CHL (1), AMC, CFM, SXT, CIP, NX, CN & CHL (1), AMC, CXM, SXT, CIP, NX, TC & CN (2)	4(3.1)	R6
AMC, CTR, CFM, SXT, CIP, NX, TC, CN & CHL (1)	1(0.8)	R7
Total Non-MDR	72(56.3)	
Total MDR	56(43.8)	
Total isolates	128 (100)	

Key: R1: Resistance to one antibiotic class; R2: Resistance to two antibiotic classes; R3: Resistance to three antibiotic classes; R4: Resistance to four antibiotic classes; R5: Resistance to five antibiotic classes; R6: Resistance to six antibiotic classes; R7: Resistance to more than six antibiotic classes, *19 isolates had a characteristic of Sensitive and Intermediate for all the selected antibiotics. Number in bracket: the number of isolates that showed resistance to the listed antibiotic classes.

Deleted[Balew Arega]: which

In cattle isolates, only 6.1% were not resistant to any of the selected antibiotics. However; 33.3% of the isolates showed Multiple Drug Resistance. From these 6.1% and 3.0% of the isolates were resistant to five and six antibiotic classes respectively (Table 7).

Deleted[Balew Arega]: s

Table 7. Multiple antibiotic resistance patterns of STEC O157:H7 isolates from cattle

Antibiotic Disk	Total Isolates N (%)	Anti-biogram
Isolates sensitive to all antibiotics (2) *	2(6.1)	R0
SXT (1), TC (3), CXM (1), CFM (1), AMC (1)	7(21.2)	R1
AMC &TC (1), SXT & CIP (2), SXT & TC (7), TC, CAZ & CTR (1), AMC, CTR & CXM (1), AMC, CTR & CFM (1)	13(39.4)	R2
AMC, TC & CN (1), AMC, CXM & TC (2)	3(9.1)	R3
AMC, TC, CN & CHL (1), AMC, SXT, TC & CN (1), AMC, CAZ, CTR, CFM, SXT & TC (1), AMC, CAZ, CFM, CXM, TC & CN (1), AMC, CAZ, CTR, CFM, CXM, SXT & TC (1)	5(15.2)	R4
AMC, CAZ, CTR, CFM, CXM, SXT, CIP, NX & TC (1), AMC, CAZ, CXM, SXT, TC & CN (1)	2(6.1)	R5

AMC, CXM, SXT, CIP, NX, TC & CN (1)	1(3.0)	R6
Total Non-MDR	22(66.7)	
Total MDR	11(33.3)	
Total isolates	33(100)	

Key: R1: Resistance to one antibiotic class; R2: Resistance to two antibiotic classes; R3: Resistance to three antibiotic classes; R4: Resistance to four antibiotic classes; R5: Resistance to five antibiotic classes; R6: Resistance to six antibiotic classes; R7: Resistance to seven antibiotic classes. * 2 isolates had a characteristic of Sensitive and Intermediate for all the selected antibiotics. Number in bracket: the number of isolates which showed resistance to the listed antibiotic classes.

Discussion

STEC O157:H7 is one cause of diarrheal disease and is mostly transmitted by cattle. Cattle are the main natural reservoirs for it (53). This study was conducted to evaluate the prevalence and antimicrobial profiles of STEC O157:H7 isolates in diarrheic patient and their cattle. Our findings showed that among 1149 diarrheic stool samples, 128 were positive for the organism. The highest prevalence was detected among under-five children. The finding also pointed out that 11.7% of isolates from diarrheic patients and 6.1% of isolates from cattle were not resistant to any of the selected antibiotics. However; Multiple Drug Resistance from the selected antibiotic classes was found in 43.8% of diarrheic patients and 33.3% of cattle.

Deleted[Balew Arega]: And t

Deleted[Balew Arega]: under

Deleted[Balew Arega]: were

The overall prevalence of STEC O157:H7 in this study for diarrheic patients was 11.1%. This was in agreement with reports from Bahir Dar, Ethiopia (13.9%) (19), and Western Kenya (11.1%) (38). However, our result was lower than those reported from Eastern Ethiopia (15.3%) (17), Eastern Cape Town, South Africa (17%) (39), and Maasai land Kenya (24.1%)

(40). On the contrary, this finding is higher than the studies reported from Gondar (1.9%) (41), Sebeta town, Ethiopia (3.2%) (14), Bishoftu town, Ethiopia (2.9%) (52), Debre Berhan cities, Ethiopia (0%) (20), Nairobi Kenya (0.2%) (42), Southern Mozambique (1.9%) (43), Morogoro Tanzania (3.6%) (44), Burkina Faso (9.67%) (45), Chattogram, Bangladesh (1.45%) (46), Sudan (5%) (60), plateau state, Nigeria (5%) (61), and France (3%) (47). The relatively higher prevalence of the organism found in the current finding might be because of a higher level of traditional breeding practice of animals and animal to human interaction in the study subjects.

In this study, STEC O157:H7 was isolated in all age groups. However, the isolation rate was high in age groups of less than 5 years i.e., 5.1% STEC O157:H7 positive from the total study population. This finding was lower than studies reported from Bahir Dar (13.9%) (19), Eastern Ethiopia (15.3%) (17), western Kenya (11.1%) (38), and Eastern Cape, South Africa (17%) (39). Even if, the result is lower than in other studies because of study population variation. In our study, under-five children were risk group for the infection. It is due to immature immune systems and poor hygiene practices among children and their caregivers at the time of food preparation, handling, transportation, and feeding.

Hand washing habit was identified as a predisposing factor for O157:H7 infection as evidenced by multivariate analysis. Patients who had an experience of hand washing after eating were about 4 times more likely to have STEC O157:H7 infections. This finding is in line with the study reported from Bahir Dar town (19).

Consuming raw vegetables, fruits, and undercooked foods is 4 times significantly associated with the prevalence of STEC O157:H7. This finding is higher than the study done in Eastern Ethiopia (17) and Bishoftu town, Ethiopia (52). This may be due to a high level of animal

Deleted[Balew Arega]: ly
Deleted[Balew Arega]: to
Deleted[Balew Arega]: under
Deleted[Balew Arega]: was
Deleted[Balew Arega]: to
Deleted[Balew Arega]: in
Deleted[Balew Arega]:
Deleted[Balew Arega]: had been
Deleted[Balew Arega]: are

manure contamination of vegetables through untreated surface water or animal manure usage as a fertilizer.

It was found that diarrheic patients who had low monthly income were 2 times more likely to have STEC O157:H7 than those who had higher monthly income. This is because income is a key instrument in applying a preventive mechanism for the predisposing factor of STEC O157:H7 infection.

Deleted[Balew Arega]: was

The prevalence of STEC O157:H7 in cattle was 14.4%. This is higher than the study done in Debre-Birhan, Ethiopia (0.81%) (20), Hawassa, Ethiopia (4.7%) (53), Jimma, Ethiopia (7.3%) (62), Addis Ababa Municipal Abattoir, Ethiopia (6.4%) (63), Debre-Zeit, Ethiopia (7%) (52), Sudan (8%) (60), and Nigeria (0.2%) (64). However, it is lower than the study reported in Iraq (91.25%) (65), and Mexico (22.03%) (54). These variations among different studies could be attributed to differences in geographical area and the type of samples taken since recto-anal mucosal swab sample is more sensitive than fecal sampling for determining the prevalence of STEC O157:H7 in ruminant animals (22).

Deleted[Balew Arega]: i

Deleted[Balew Arega]: ,

The availability of cattle in the diarrheic patient house was about 3 times more likely to have STEC O157:H7 compared to the counterpart. This finding is in line with the report in Eastern Ethiopia (17), and Harare, Zimbabwe (56). However, it was lower than the report from Germany (57). The ruminant, especially cattle had been identified as the major reservoir of STEC O157:H7, there could be cross-infection to the diarrheic patients through either direct live animal contact or with animal manure. Animal manure can contaminate food, water, and the environment, and some studies reported that contact with cattle and living in or visiting a place with farm animals are risk factors for the incidence of STEC O157:H7 infection (58).

In this study, STEC O157:H7 isolates (n=128) in diarrheic patients showed antibiotic resistance at varying degrees. Of all the antimicrobials tested, the highest resistance was found in tetracycline 70(54.7%), and Amoxicillin/Clavulanate 68(53,1%). Among the STEC O157:H7 isolates from cattle RAMS, the highest resistances were found in tetracycline 23(69.7%), Sulfamethoxazole/Trimethoprim 16(48.5%), and Amoxicillin/clavulanate 15(45.4%). This finding is in agreement with the report in Bahir Dar (19) but is indifferent to Cape Town (55). This difference may be due to differences in the practice of prescribing the selected antibiotic between the two countries and even the practice of misuse of antibiotics.

Deleted[Balew Arega]:

Even if, the place of defecation, level of contamination of the water source, and sharing of home with cattle were not found associated with the occurrence of STEC O157:H7. However, other researchers reported open field defecation, drinking contaminated water sources, the presence of the diarrheic person in the house, and the use of common house with ruminants including cattle as a predictor for the occurrence of STEC O157:H7 (17, 51, 59). The detection of STEC O157:H7 in human and their cattle calls for further epidemiological assessment to detect the source of infection, and means of transmission, and follow a prevention system.

As to the limitations of this study, we have focused only on STEC O157:H7 strain, but other pathogenic non-STEC O157:H7 serogroups causing illnesses in human needs to be identified.

Conclusion

The present study revealed a high occurrence of STEC O157:H7 in diarrheic patients and their cattle. Age, patients with bloody diarrhea, the presence of cattle in their house, the presence of another diarrheic person in the house, and the habit of consuming raw foods were more important risk factors for STEC O157:H7 infections than their counterparts. The study also

pointed out the presence of resistant STEC O157:H7 to one or more antimicrobials that may lead to a potential public threat. Generally, this study could give an insight into the prevalence of STECO157:H7 and its public health significance associated with contact cattle, consumption of raw food, and poor personal hygienic practices. Therefore, special attention and awareness should be given to teaching the community how to give care to ruminant animals and their home, proper sanitation measures for raw foods, and hand washing habits before preparing food, before eating, and after giving care to patients, and animals. Moreover, medical professionals and veterinarians should work together to give health education to the public concerning STEC O157:H7.

Deleted[Balew Arega]: teach

Deleted[Balew Arega]: on

Deleted[Balew Arega]: with respect to

Supporting information

S1 File. Laboratory Standard Operational Procedure (SOP) (media used, laboratory procedures, and antimicrobial disks used for their interpretation) (DOCX)

Acknowledgment

We would like to thank the University of Gondar for covering the data collection fee and purchase of laboratory materials /reagents for the project. All of the study participants, data collectors, and other staff at the respective hospitals are acknowledged for their cooperation during the data collection processes.

Funding

The authors received no specific funding for this work.

Conflict of interest

The author declares that there is no competing interest.

Author contribution

Conceptualization: Tigist Engda, Professor Feleke Moges, Professor Belay Tessema

Data curation: Tigist Engda, Professor Feleke Moges, Professor Belay Tessema, Dr. Nebiyu Mesfin, Dr. Anwar Nuru, Teshome Belachew

Formal analysis: Tigist Engda, Professor Feleke Moges

Investigation: Tigist Engda

Methodology: Tigist Engda. Professor Feleke Moges Professor Belay Tessema, Dr. Nebiyu Mesfin, Dr. Anwar Nuru, Teshome Belachew

Project administration: Tigist Engda

Resources: Tigist Engda

Software: Figist Engda, Professor Feleke Moges, Professor Belay Tessema, Dr. Nebiyu Mesfin

Supervision: Tigist Engda

Validation: Tigist Engda, Professor Feleke Moges, Professor Belay Tessema, Dr. Nebiyu Mesfin, Dr. Anwar Nuru, Teshome Belachew

Visualization: Tigist Engda, Professor Feleke Moges

Writing – original draft: Tigist Engda

Writing – review & editing: Tigist Engda, Professor Feleke Moges, Professor Belay Tessema, Dr. Nebiyu Mesfin, Dr. Anwar Nuru

References

1. Naghavi M, Abajobir AA, Abbafati C, Abbas KM, Abd-Allah F, Abera SF, et al. Global, Regional, and National Age-Sex specific Mortality for 264 Causes of Death, 1980-2016: A Systematic Analysis for the Global Burden of Disease Study 2016. *Lancet.* 2017; 390(10100):1151–210. 10.1016/S0140-6736(17)32152-9.

2. Baale E. Determinants of diarrhea and acute respiratory infection among under-fives in Uganda. *Australas Med J*. 2011; 4: 400-407. PMID: 23393526, PMCID: PMC3562942, DOI: 10.4066/AMJ.2011.723.

3. United Nations Children's Fund (UNICEF), World Health Organization. Diarrhea. Why Children are Dying and What Can be done? New York, NY: UNICEF. 2009; ISBN 978-92-806-4462-3 (UNICEF).

4. IVAC. Pneumonia & Diarrhea Progress Report 2020. International Vaccine Access Center (IVAC). 2020 [Available from: https://resourcecentre.savethechildren.net/pdf/ivac-pdpr-2020.pdf/

5. Ryan U, Zahedi A, Paparini A. Cryptosporidium in humans and animals-a one health approach to prophylaxis. *Parasite Immunol*. 2016; 38(9): 535–47. PMID: 27454991 DOI: 10.1111/pim.12350.

6. Havelaar AH, Kirk MD, Torgerson PR, Gibb HJ, Hald T, Lake RJ, et al. World Health Organization Global Estimates and Regional Comparisons of the Burden of Foodborne Disease in 2010. *PLoS Med*. 2015; 3:12(12). http://doi.org/10.1371/journal. Pmed.1001923.

7. Croxen MA, Law RJ, Scholz R, Keeney KM, Wlodarska M, Finlay BB. Recent advances in understanding enteric pathogenic Escherichia coli. *Clin Microbiol Rev*. 2013; 26: 822–880. PMID: 24092857, PMCID: PMC3811233, DOI: 10.1128/CMR.00022-13.

8. Croxen MA, Law RJ, Scholz R, Keeney KM, Wlodarska M, Finlay BB. Recent advances in understanding enteric pathogenic Escherichia coli. *Clin Microbiol Rev*. 2013; 26:822–80. PMID: 24092857, PMCID: PMC3811233, doi: 10.1128/CMR.00022-13.

9. Hickey CA, Beattie TJ, Cowieson J, MiyashitaY, Strife CF, Frem JC, Peterson JM et al. Early volume expansion during diarrhea and relative nephroprotection during subsequent

hemolytic uremic syndrome. *Arch. Pediatr. Adolesc.Med.* 2011; 165 (10): 884-889. PMID: 21784993, PMCID: PMC4064458, DOI: 10.1001/archpediatrics.2011.152.

10. Ji Youn Lim, Jang W. Yoon, and Carolyn J. Hovde. A Brief Overview of *Escherichia coli* O157:H7 and Its Plasmid O157. *J Microbiol Biotechnol.* 2010; 20(1): 5–14. PMCID: PMC 3645889, PMID:20134227, NHMSID: NHMS464337.

11. Lal et al., Lal et al. Seasonality in human zoonotic enteric diseases: a systematic review. *PLoS ONE*. 2012; 7 (4), https://doi.org/10.1371/journal.pone.0031883.

12. Launders N, Byrne L, Jenkins C, Harker K, Charlett A, Adak GK. Disease severity of Shiga toxin-producing E. coli O157 and factors influencing the development of typical haemolytic uraemic syndrome: a retrospective cohort study, 2009-2012. *BMJ*. 2016; Jan 29;6(1), *PMID: 26826153, PMCID: PMC4735319, DOI: 10.1136/bmjopen-2015-009933.*

13. Amézquita-López B.A., Soto-Beltrán M., Lee B.G., Yambao J.C., Quiñones B. Isolation, genotyping and antimicrobial resistance of Shiga toxin-producing Escherichia coli. J. *Microbiol Immunol Infect.* 2018; 51:425–434. PMID: 28778595, doi: 10.1016/j.jmii.2017.07.004.

14. Demis B W. Occurrence and antimicrobial resistance of E. coli O157:H7 in cattle, beef and diarrheic patients in Sebeta town, Ethiopia. MSc thesis. Addis Ababa university, college of veterinary medicine and agriculture, department of microbiology, immunology, and veterinary public health, Bishoftu, Ethiopia, 2020.

15. Raies A. Mir, Brian W. Brunelle, David P. Alt, Terrance M. Arthur, and Indira T Kudva. Supershed Escherichia coli O157:H7 Has Potential for Increased Persistence on the Rectoanal Junction Squamous Epithelial Cells and Antibiotic Resistance. *Int J Microbiol. 2020;* ID 2368154 | https://doi.org/10.1155/2020/2368154.

16. Segura A, Auffret P, Bibbal D, Bertoni M, Durand A, Jubelin G. et al. Factors Involved in the Persistence of a Shiga Toxin-Producing *Escherichia coli* O157:H7 Strain in Bovine Feces and Gastro-Intestinal Content. *Front Microbiol.* 2018; 9: 375, PMID: 29593666, PMCID: PMC5854682, doi: 10.3389/fmicb.2018.00375.

17. Getaneh D K, Hordofa L O, Ayana DA, Tessema T S, Regassa L D. Prevalence of *Escherichia coli* O157:H7 and associated factors in under-five children in Eastern Ethiopia. *PLOS ONE*. 2021; 16(1): e0246024. https://doi.org/10.1371/journal.pone.0246024.

18. Gutema FD, Rasschaert G, Agga GE, Jufare A, Duguma AB, Abdi RD. et al. Occurrence, Molecular Characteristics, and Antimicrobial Resistance of Escherichia coli O157 in Cattle, Beef, and Humans in Bishoftu Town, Central Ethiopia. *Foodborne Pathog Dis*. 2021 ;18(1):1-7. PMID: 32865441, DOI: 10.1089/fpd.2020.2830.

19. Adugna A, Kibret M, Abera B, Nibret E, and Adal M. Antibiogram of E. coli serotypes isolated from children aged under five with acute diarrhea in Bahir Dar town. *Afr Health Sci.* 2015; 15(2): 656–664, PMID: 26124817, DOI 10.4314/ahs.v15i2.45.

20. Abdissa R, Haile W, Fite A T, Beyi A F, Agga G E, Edao et al. Prevalence of *Escherichia coli* O157:H7 in beef cattle at slaughter and beef carcasses at retail shops in Debre Berhan cities, Ethiopia. *BMC Infect Dis*, 2017; 17:277. https://doi.org/10.1186/s12879-017-2372-2.

21. Central Statistical Agency of Ethiopia. Population Projection of Ethiopia for All Regions at Woreda Level from 2014 – 2017. *Federal Democratic Republic of Ethiopia Central Statistical Agency, Addis Ababa.* SCIRP. 2013; 4-38.

22. A. H. Havelaar, M. D. Kirk, P. R. Torgerson et al., "World health organization global estimates and regional comparisons of the burden of foodborne disease in 2010," PLoS Medicine. 2015; 12(12).

23. Michael Bording-Jorgensen, Hannah Tyrrell, Colin Lloyd, and Linda Chui. Comparison of Common Enrichment Broths Used in Diagnostic Laboratories for Shiga Toxin-Producing *Escherichia coli. Microorganisms.* 2021; 9(3), 503. https://doi.org/10.3390/microorganisms9030503.

24. Mcpherson A S, Dhungyel O P and Ward M P. Comparison of recto-anal mucosal swab and faecal culture for the detection of Escherichia coli O157 and identification of super-shedding in a mob of Merino sheep. *Epidemiol. Infect.* 2015; 143, 2733–2742, doi:10.1017/S0950268815000011

25. Williams I, Richardson J, Starkey A, Stansfield I. Genome-wide prediction of stop codon readthrough during translation in the yeast Saccharomyces cerevisiae. *Nucleic Acids Res*. 2014; 32(22): 6605-16, PMID: 15602002, PMCID: PMC545446, DOI: 10.1093/nar/gkh1004.

26. Quinn PJ, Markey BK, Leonard FC, Hartigan P, Fanning S, E. F. Veterinary microbiology and microbial disease. *Sons; JW, editor.* 2011; ISBN: 978-1-405-15823-7.

27. Sagar, A. Bacterial Identification: Biochemical Test and Identification of E. coli. *Online Microbiology Notes*. 2015. URL: *https://microbiologyinfo.com/biochemical test-and-identification-of-e-coli/. Accessed on 2017.*

28. Aklilu, M., Sisay, T., Tefera, G. and Tekalign, B. Identification and biotyping of *Escherichia coli* from diarrheic lambs in and around Debre Birhan town, Ethiopia. J. *Environ. Anal. Toxicol*. 2013; 3:188.

29. Carroll AM, Cobban E, McNamara EB. Evaluation of molecular and culture methods to determine the optimum testing strategy for verotoxigenic Escherichia coli in faecal specimens. *Diagnostic Microbiology and Infectious Disease*. 2016; 85(1):1–5. PMID: 26830271, DOI: 10.1016/j.diagmicrobio.2015.12.011.

30. Remel RIM™ E. coli O157:H7 Latex Test kit, IFU24250, Revised July, 2020. www.thermofsher.com/microbiology.

31. Iwu CJ, Jaja IF, Iweriebor BC, Obi LC, Okoh AI. “Antibiotic resistance profiles of *Escherichia coli* O26, O145, and O157: H7 isolated from swine in the eastern Cape Province, South Africa.” *Asian Pacific Journal of Tropical Disease*. 2017; 7 (9): 553–559. DOI:10.12980/apjtd.7.2017D7-9.

32. Ningrum S G, Soejoedono R D, Latif H, Arnafia W, & Wibawan I W T. Prevalence and Characterization of Shiga Toxin-Producing *Escherichia coli* Isolated from Slaughtered Qurban Animal in Jakarta Province. *Media Peternakan*. 2016; 39(2):90-94 DOI: 10.5398/medpet.2016.39.2.90 Available online at http://medpet.journal.ipb.ac.id.

33. Imtiaz j, Hashmi I, Saeed A, Qazi I A & Arshad M. Development of PCR protocol for detection of Escherichia coli in drinking water. *WIT Transactions on Ecology and The Environment*. 2013; 171(10): 225-234.DOI: 10.2495/WRM130201.

34. Mohmmed H. Khudor, Awatif H. Issa, and Farooq L. Jassim. Detection of rfb O157 and fliCH7 Genes in *Escherichia coli* Isolated from Humans and Sheep in Basrah Province. Raf J. Sci. 2012; 23(1):19-33.

35. Clinical and Laboratory Standards Institute. Performance standards for antimicrobial susceptibility testing. 32nd ed. CLSI supplement M100. Wayne: Clinical and Laboratory Standards Institute. 2022; 42(2).

36. Reza RH, Shipa SA, Naser MN, Miah F. Surveillance of *Escherichia coli* in a fish farm of Sylhet, Bangladesh. *Bangladesh J Zool*. 2021; 48(1): 335–346. DOI: https://doi.org/10.3329/bjz.v48i2.52373.

37. Tadesse HA, Gidey NB, Workelule K, Hailu H, Gidey S, Bisrat A, et al. Antimicrobial resistance profile of E. coli isolated from raw cow milk and fresh fruit juice in Mekelle, Tigray, Ethiopia. Vet Med Int. 2018; PMID: 29755727 PMCID: PMC5884018, DOI: 10.1155/2018/8903142.

38. Rono SJ, Kakai R, Esamai F, Mibei E, Odundo E. Pathotypes and virulence markers in *Escherichia coli* associated with diarrhea among HIV seropositive and seronegative children below five years in western Kenya. *European Scientific Journal*. 2014; 10 (27): 1857 – 7881. DOI: https://doi.org/10.19044/esj.2014.v10n27p%25p.

39. Omolajaiye S A, Afolabi K O, and Iweriebor B C. Pathotyping and Antibiotic Resistance Profiling of Escherichia coli Isolates from Children with Acute Diarrhea in Amatole District Municipality of Eastern Cape, South Africa. *Biomed Res Int*. 2020. PMID: 33294442, PMCID: PMC7691003, DOI: 10.1155/2020/4250165.

40. Sang WK, Boga HI, Waiyaki PG, Schnabel D, Wamae NC, Kariuki SM. prevalence and genetic characteristics of Shigatoxigenic Escherichia coli from patients with diarrhoea in Maasailand, Kenya. *Journal of Infection in Developing Countries*, 2012, 6(2). DOI: https://doi.org/10.3855/jidc.1750.

41. Getie M, Abebe W, Tessema B. Prevalence of enteric bacteria and their antimicrobial susceptibility patterns among food handlers in Gondar town, Northwest Ethiopia. *Antimicrobial Resistance and Infection Control*. 2019; 8:111, https://doi.org /10.1186/s13756-019-0566-7.

42. Onyango A O, Kenya E U, Mbithi J N, Ng'ayo M O. Pathogenic *Escherichia coli* and food handlers in luxury hotels in Nairobi, Kenya. *Travel Medicine and Infectious Disease*. 2009; 7: 359 – 366. PMID: 19945013, DOI: 10.1016/j.tmaid.2009.07.005.

43. Mandomando I M, Macete E V, Ruiz J, Sanz S, Abacassamo F, Vallès X. et al. Etiology of diarrhea in children younger than 5 years of age admitted in a rural hospital of southern Mozambique. Am J Trop Med Hyg. 2008; 79(1):140. PMID: 17360878.

44. Raji1 M A, Minga U M and Machang'u R S. Prevalence and characterization of verotocytoxin producing Escherichia coli O157 from diarrhea patients in Morogoro, Tanzania. *Tanzania Journal of Health Research*. 2008; 10 (3). PMID: 19024340 DOI: 10.4314/thrb. v10i3.14355.

45. Konaté A, Dembélé R, Kagambèga A, Soulama I, Kaboré WA D, Sampo E, et al. Molecular characterization of diarrheagenic Escherichia coli in children less than 5 years of age with diarrhea in Ouagadougou, *Burkina faso. Eur j Microbiol Immunol*. 2017; 7(3): 220–228. DOI: 10.1556/1886.2017.00011.

46. Mohammad Sharif Uddin, Md. Mijanur Rahman , Md. Omar Faruk , Asma Talukder, Md. Imranul Hoq , Suman Das, and K. M. Shariful Islam. Bacterial gastroenteritis in children below five years of age: a cross-sectional study focused on etiology and drug resistance of Escherichia coli O157, Salmonella spp., and Shigella spp. *Bull Natl Res Cent*. 2021; 45:138 https://doi.org/10.1186/s42269-021-00597.

47. Pradel N, Livrell V, Champs C De, Palcoux JB, Reynaud A, Scheutz F. Prevalence and characterization of Shiga-toxin producing Escherichia coli isolated from cattle, food and children during a one-year prospective study in France. *J Clin Microbiol*. 2000; 38: 1023-1031. PMID: 10698990, PMCID: PMC86328, DOI: 10.1128/JCM.38.3.1023-1031.2000.

48. Ohaiseadha O C, Hynds PD, Fallon UB, O'Dwyer J. A geostatistical investigation of agricultural and infrastructural risk factors associated with primary verotoxigenic E. coli (VTEC) infection in the Republic of Ireland, 2008–2013. *Epidemiology and infection*. 2017; 145(1): 95–105. PMID:27609320, PMCID: PMC9507320, DOI:10.1017/S095026881600193X.

49. Byrne L, Jenkins C, Launders N, Elson R, Adak GK. The epidemiology, microbiology and clinical impact of Shiga toxin-producing *Escherichia col*i in England, 2009–2012. *Epidemiology and infection.* 2015; 143(16):3475–87. PMID: 25920912, PMCID: PMC9150938, DOI: 10.1017/S0950268815000746.

50. McKee RS, Tarr PI, Dietzen DJ, Chawla R, Schnadower D. Clinical and Laboratory Predictors of Shiga Toxin-Producing Escherichia coli Infection in Children with Bloody Diarrhea. *Journal of the Pediatric Infectious Diseases Society*. 2018; 7(3). PMID: 29617871, PMCID: PMC6826345, DOI: 10.1093/jpids/piy025.

51. Treacy J, Jenkins C, Paranthaman K, Jorgensen F, Mueller-Doblies D, Anjum M, et al. Outbreak of Shiga toxin-producing Escherichia coli O157:H7 linked to raw drinking milk resolved by rapid application of advanced pathogen characterization methods, England, August to October 2017. *European communicable disease bulletin*. 2019; 24(16): 1800191. PMID 31014418, DOI: 10.2807/1560-7917.ES.2019.24.16.1800191.

52. Jufare, A. Occurrence and Antimicrobial susceptibility profile among E. coli 0157:H7 isolated from the Abattoir, retail shop and diarrheic patient in Bishoftu town, East Shoa Ethiopia. MSc Thesis, Addis Ababa University, Faculty of Veterinary Medicine, Debre-Zeit, Ethiopia, 2018.

53. Atnafie B, Paulos D, Abera M, Tefera G, Hailu D, Kasaye S, Amenu K. Occurrence of Escherichia coli O157:H7 in cattle feces and contamination of carcass and various contact surfaces in abattoir and butcher shops of Hawassa, Ethiopia. *BMC Microbiol*. 2017; 17(1): 24. PMID: 28122502, PMCID: PMC5264334, DOI: 10.1186/s12866-017-0938-1.
54. Jos Lara-Duran JA; Vega MS; Valenzuela R B, Ruiz C D, Ruiz OD. Incidence of Escherichia coli 0157:H7 in feces of lactating ruminants with diarrheal syndrome. *Journal MVZ Cordoba*. 2019; 24(3):7339-7345. https://doi.org/10.21897/rmvz.1232.
55. Kalule JB, Keddy KH, and Nicoll MP. Characterization of STEC and other diarrheic E. coli isolated on CHROMagar™ STEC at a tertiary referral hospital, Cape Town John. *BMC Microbiology*. 2018; 18:5. https://doi.org/10.1186/s12866-018-1195-7.
56. Navab-Daneshmand T, Friedrich MND, Gachter M, Montealegre MC, Mlambo LS, Nhiwatiwa T, et al. Escherichia coli Contamination across Multiple Environmental Compartments (Soil, Hands, Drinking Water, and Handwashing Water) in Urban Harare: Correlations and Risk Factors. *The American journal of tropical medicine and hygiene*. 2018; 98(3):803–13. PMID: 29363444, PMCID: PMC5930891, DOI: 10.4269/ajtmh.17-0521.
57. Werber D, Behnke SC, Fruth A, Merle R, Menzler S, Glaser S, et al. Shiga toxin-producing Escherichia coli infection in Germany- different risk factors for different age groups *Am J Epidemiol.* 2007; 165(4):425-34. PMID: 17158472, DOI: 10.1093/aje/kwk023.
58. Friesema IH, Schotsborg M, Heck ME, Van Pelt W. Risk factors for sporadic Shiga toxin-producing Escherichia coli O157 and non-O157 illness in the Netherlands, 2008–2012,

using periodically surveyed controls. *Epidemiology and infection*. 2015; 143(7):1360–7. PMID: 25195737, PMCID: PMC9507206, DOI: 10.1017/S0950268814002349.

59. Abong'o BO, Momba MN. Prevalence and potential link between E. coli O157:H7 isolated from drinking water, meat and vegetables, and stools of diarrheic confirmed and non-confirmed HIV/AIDS patients in the Amathole District—South Africa. *Journal of applied microbiology*. 2008; 105(2):424–31. PMID: 18298530, DOI: 10.1111/j.1365-2672.2008. 03756.x.

60. Musa, H.A., Shikieri, A.B., Ahmed, H.H. and Kafi, S.K. Isolation and identification of E. coli 0157:H7 amongst Sudanese patients with bloody diarrhea and in animal. *Sudan JMS*., 2010, 5(2): 91-94.

61. Itelima, J. U., Agina, S.E., Ogbonna, A. I. and Nwaukwu, I. A. The Occurrence of Escherichia Coli Serotpye O157: H7 among Humans in Some Parts Plateau State, Nigeria. *DMMB*. 2014; 5(1): 9-20.

62. Haile, A.F., Kebede, D. and Wubshet, A.K. Prevalence and antibiogram of Escherichia coli O157 isolated from bovine in Jimma, Ethiopia: abattoir-based survey. *Ethiop. Vet. J.*. 2017; 21(2): 109-120.

63. Hamid, M., Tefera, Y., Eguale, T. and Worku, Y. Escherichia coli O157:H7: Prevalence, Identification, and Antimicrobial Resistance in Cattle Slaughter at Addis Ababa Municipal Abattior, Ethiopia. *Int. J. Adv. Res. Biol. Sci*. 2018; 5(10): 136-146.

64. Mailafia, S., Madubuike, S. A.1., Raji, M. A., Suleiman, M.M., Olabode, H.O. K., Echioda Egbole, M. and Okoh, G. P. R. Phenotypic identification of Escherichia coli O157:H7 isolates from cattle at Suleja Abattoir, Nigeria. *Afr. J. Microbiol. Res*. 2017; 11(21): 845-850.

65. Al-Rudha, A.M.H., Al-Rubaie, E.M. and Khalil, N.K. Distribution of E. coli O157:H7 in fecal and urine samples of cattle. *Iraqi. J. Vet. Med.*, 2016; 40(1): 79-82.
