# Supplementary material for: Shiga toxin-producing Escherichia coli O157:H7 among diarrheic patients and their cattle in Amhara National Regional State, Ethiopia
Source: PLoS One. 2023 Dec 21;18(12):e0295266. doi: 10.1371/journal.pone.0295266 (PMC10734908; doi:10.1371/journal.pone.0295266)
Supplement: Attachment — Submitted filename: plose one review document.doc [file pone.0295266.s002.doc]

**Comments to Authors:**

This is an interesting study reflecting the STEC O157:H7 in the study region and may have good input for scientific people in the area of interest. The authors conducted an extensive study, collected a large number of samples, and carried out scientifically sound bacterial assessment. However, the write up and improving the following comments and suggestions is good.

1. Did the author clarify: Page 2 line number 29 ………………………………….Ethiopia from December/2020 to May/2022. Page 4 line 89………………….from December 2020 to June 2022. Which one was the exact study period?
2. Line 89: study was carried out……. from December/2020 to May/2022. A total 1,149 Diarrheic stool samples and 229 cattle Recto-anal mucosal swab samples were collected.” The samples were collected over one year, meaning at different seasons of the year. Did you consider the effects of season on the disease level?
3. The description of the sampling strategy is not clear. Page 6, line 116 Authors wrote that ……………The study participants were selected using simple random sampling techniques. How could be? Because the study is institution based; that means diarrheic patients is not known their name before visited the hospital. Or may not wait you after they gain service they leave the hospital except admit ones. So, how can list their name and lottery then sample? Can Authors clarify? I think you mean systematic random sampling????
4. Page 6 line numbers 118-120; Twelve-month diarrheic cases were taken from patient registration book in order to project the probable case flow in each hospital per day. Then the study subjects were recruited consecutively until the desired sample was reached. Is not clear; Can Authors elaborate in figure/number to clarify the readers?
5. In the case of cattle, convenient sampling techniques were used. All cattle found in STEC O157:H7-positive houses were included in the study and were found 229. Not clear; do you mean convenient sampling? Or census/all cattle sample?

- Since hospital service users come from distance even from politically unstable areas; so, how do you trace back to their cattle to include all cattle in positive houses? E.g. Beshangule gumize, Jawi to Felghiwote hospital, Afar region to Dessie hospital etc…

6. A scholar suggests???? *E.coli* is may originated from human, other animal feces not only cattle.” I’m afraid how to handle other source of infection other than cattle. E.g. sheep, dogs, other non-diarrheic humans etc…
7. Page 12 line 257…………..categorical variables, univariable and multivariate analysis with a 95% confidence interval (CI)………………change by categorical variables, bi-variable and multivariable logistic regression analysis with 95% confidence interval(CI)……………………. Because to make uniformity; and multivariate analysis and multivariable analysis are quite different.
8. Page 29 your conclusion part line # 421-422 Shiga toxin-producing *E. coli* O157:H7 isolates developed drug resistance to most antimicrobials tested. Giving antibiotic treatment in *E.coli* infection of human and animal cause’s worse of the disease thus not recommended; despite this, where is the drug resistance come from?
9. In addition u tested number of antibiotics those are ban and not used in animals? E.g. Chloramphenicol; what is the reason for the discrepancy?
