# Supplementary material for: Shiga toxin-producing Escherichia coli O157:H7 among diarrheic patients and their cattle in Amhara National Regional State, Ethiopia
Source: PLoS One. 2023 Dec 21;18(12):e0295266. doi: 10.1371/journal.pone.0295266 (PMC10734908; doi:10.1371/journal.pone.0295266)
Supplement: Attachment — Submitted filename: Response to Reviewers.docx [file pone.0295266.s003.docx]

Date: August1, 2023

**To:** Plose one

Editor-In-Chief

**From:** Tigist Engda (PI and corresponding author)

**Submission ID**: PONE-D-23-13678

**Title:** Shiga toxin-producing Escherichia coli O157:H7 among diarrheic patients and their cattle in Amhara National Regional State, Ethiopia

**Point by point authors’ response**

First, we would like to thank and appreciate the reviewers for their critical and constructive comments. We attempted all the questions and concerns raised by chief reviewers point by point as follows:

**I.** **JOURNAL REQUIREMENTS:**

1. Please ensure that your manuscript meets PLOS ONE's style requirements, including those for file naming.

**Author response:** Thank you. We have followed all the PLOS One requirements**.**

2**.** Your ethics statement should only appear in the Methods section of your manuscript.

**Author response**: Thank you. We shifted the ethics statement to the Method section as recommended and deleted it from the other section.

3. PLOS ONE now requires that authors provide the original uncropped and unadjusted images underlying all blot or gel results reported in a submission’s figures or Supporting Information files.

**Author response:** Thank you. We replace the clear image as requested

**II. REVIEWERS COMMENTS**

**Comments for Reviewer I.**

1. **Comment**: Study population needs clarification. It seems similar with that of source population.

**Authors response:** Thank you! We have noted and explained as the source population is all diarrheic patients who had visited the respective Comprehensive Specialized Hospitals for medical services and their cattle. However; Those diarrheic patients who are volunteers for the study and fulfilled the requirements to be the study participants (giving informed consent and the required amount of stool samples for laboratory analysis) are the study population. Page5, line 102-105.

2. **Comment:** Exclusion criteria needs revision. The authors said they excluded patients who has been on antibiotic treatment at the time of data collection but realistically they should have excluded those who are on antibiotic treatment for the last 7 days too.

**Authors response**: Thank you! Yes of course; those who had taken antibiotics during the past 7 days of data collection were excluded but might be cut during the editing of the document therefore we are now corrected. Page5, line112-114.

3. **Comment**: How antibiotics used for antimicrobial susceptibility testing were selected?

**Authors response:** Thank you! It is a good comment, it is selected based on the Clinical and Laboratory Standards Institute (CLSI, 2022) guideline. All the selected drugs were effective for diarrheal diseases caused by E. coli. However, we include norfloxacin which can be used as an effective drug for the treatment of diarrheagenic E. coli (*Msolo Luyanda; et al; 2020*). In other selected drugs, there is at least one drug frequently prescribed to treat diarrheal diseases caused by bacteria in their class, so we are trying to check the resistance rate of drugs in each class of antimicrobials. (Ref: “*Msolo L, Iweriebor B C, Okoh A I.* *Antimicrobial Resistance Profiles of Diarrheagenic E. coli (DEC) and Salmonella Species Recovered from Diarrheal Patients in Selected Rural Communities of the Amathole District Municipality, Eastern Cape Province, South Africa. Dovepress journal Infection and Drug Resistance, 2020:13 4615–4626”).*

4. **Comment**: Where did you classify Intermediate susceptibility during data analysis? (Is it susceptible or resistant?)

**Authors response:** Thank you for the concern. Intermediate susceptibility was small in number and included as sensitive.

5. **Comment:** What controls did you use for PCR?

**Authors response:** Thank you! We have noted and corrected it as ‘‘A negative control was performed by adding 10 µl of sterile deionized water; a positive control was performed by adding 1.25µl of known DNA sample (STEC O157:H7; ATCC 43895)’’. Page 10, lines 212-214

6. **Comment**: The agarose gel electrophoresis image is not clear (please replace it with a clear image).

**Authors response**: Thank you! for the comment, it has been replaced by a clear image.

7. **Comment**: The prevalence, risk factors, and drug resistance patterns discussion was not done exhaustively (fewer literature were used for comparison purposes), and the reasons given for variations of your findings with other research seems similar throughout the discussion.

**Authors response:** Thank you! Even though there are not much more studies done in our country for comparison. We are now revised.

8. **Comment**: You mentioned "Even if, the place of defecation, level of contamination of the water source, and sharing of home with cattle were not found associated with the occurrence of STEC O157:H7. However, other researchers reported it." what do you think are the reasons for this? You should explain in the discussion section.

**Author response**: Thank you! for your logical question. First of all, when we see the socio-demographical characteristics of the study population, only 25.7% of STEC O157: H7 positive patients shared their home with cattle, and the remaining (74%) do not. From this data, we cannot expect an association. However, in studies were higher results of sharing a home with cattle, there may be an association and the reason can work. Similarly, in surface water sources and open field defecation was detected in 3.9% and 22.7% of STEC O157:H7 positive respectively, and has no association. This may be due to less positive isolate.

**9. Comment:** In the Conclusion section you mentioned "...teaching the community." do you mean health education?

**Author response:** Yes. It means health education.

10. **Comment**: Some references are very old (Gannon, 1997; Slutsker, 1998; Olorunshola, 2000; Zhang, 2000; Pruimboom-Brees, 2000; Raji, 2003; Naylor, 2003; Kang, 2004; Thrusfield, 2005; Bryce, 2005; Casburn-Jones, 2004; Cheesbrough, 2006; Brenner, 2005) and MacFaddin JF,2000. Please replace them with the latest literature.

**Author response:** Thank you. The comment is accepted and replaced by other references.

**Comments for Reviewer II.**

This is an interesting study reflecting the STEC O157:H7 in the study region and may have good input for scientific people in the area of interest. The authors conducted an extensive study, collected a large number of samples, and carried out scientifically sound bacterial assessments. However, the write-up and improving the following comments and suggestions is good.

**1. Comment:** Did the author clarify: Page 2 line number 29…………………Ethiopia from December/2020 to May/2022. Page 4 line 89……………from December 2020 to June 2022. Which one was the exact study period?

**Authors response:** Thank you for the comment. we have noted and the correct one is December 2020 to June 2022. So, it is corrected in the text as’’ December 2020 to June 2022’’.

**2. Comment**: Line 89: study was carried out……. from December/2020 to May/2022. A total of 1,149 Diarrheic stool samples and 229 cattle Recto-anal mucosal swab samples were collected.” The samples were collected over one year, meaning at different seasons of the year. Did you consider the effects of season on the disease level?

**Author’s response:** Yes, they are different since around half of human cases are sporadic with a seasonal pattern favoring spring and summer. Also, in this study, 49.2% and 25.8% of STEC O157:H7 isolate was detected in the summer and spring seasons respectively.

**3. Comment:** The description of the sampling strategy is not clear. Page 6, line 116 Authors wrote that …………… The study participants were selected using simple random sampling techniques. How could be? Because the study is an institution based; that means diarrheic patients is not known their names before visiting the hospital. Or may not wait for you after they gain service, they leave the hospital except admit ones. So, how can list their name and lottery then sample? Can Authors clarify? I think you mean systematic random sampling????

**Author’s response:** Yes, the comment is well accepted and corrected as” A simple random sampling technique was used to select Comprehensive Specialized Hospitals. However, the study participants were selected using systematic random sampling techniques”. Page6, line 122and 121.

**4. Comment**: Page 6 line numbers 118-120; Twelve-month diarrheic cases were taken from the patient registration book in order to project t2e probable case flow in each hospital per day. Then the study subjects were recruited consecutively until the desired sample was reached. Is not clear; Can Authors elaborate in figures/numbers to clarify the readers?

**Authors response:** Thank you! We accepted the comment and corrected it on page 6, lines 122-136.

**5. Comment**: In the case of cattle, convenient sampling techniques were used. All cattle found in STEC O157:H7-positive houses were included in the study and were found 229. Not clear; do you mean convenient sampling? Or census/all cattle sample?

- Since hospital service users come from a distance even from politically unstable areas; so, how do you trace back to their cattle to include all cattle in positive houses? E.g. Beshangule gumize, Jawi to Felghiwote hospital, Afar region to Dessie hospital etc…

**Authors response:** Yes. the comments are well accepted and corrected as ‘’all cattle found in the STEC O157:H7 positive houses were included in the study.’’ Page 6, lines 132 to 134. Of course, geographical distribution (highly remote areas), and politically unstable areas are challenges to achieved the plan of sample collection. However, in our study, the cattle used for sample collection are around the selected hospital. this means; it is not as far or remote. In addition; the specific house was found with the help of animal science personnel at the site and registered STEC O157:H7 positive diarrheic patient profile (Name, phone no., specific name of the site (kebele name, ‘Gote’ name)).

**6. Comment:** A scholar suggests???? E. coli is may originated from human, or other animal feces not only cattle.” I’m afraid how to handle another source of infection other than cattle. E.g. sheep, dogs, other non-diarrheic humans etc…

**Authors response:** Thank you! of course, animals, other than cattle and asymptomatic humans (non-diarrheic humans) can be the source of STEC O157:H7. So, to manage the distribution of STEC O157:H7, we have to prevent the root of transmission by eating cooked and non-contaminated food, having proper contact with animals and also human

**7. Comment:** Page 12 line 257…………..categorical variables, univariable and multivariate analysis with a 95% confidence interval (CI)………………change by categorical variables, bi-variable and multivariable logistic regression analysis with 95% confidence interval(CI)……………………. Because to make uniformity, and multivariate analysis and multivariable analysis are quite different.

**Author’s Response:** Yes, multivariate analysis and multivariable analysis are completely different and the comment is well taken and corrected in the main document.

**8. Comment:** Page 29 your conclusion part line # 421-422 Shiga toxin-producing E. coli O157:H7 isolates developed drug resistance to most antimicrobials tested. Giving antibiotic treatment in E.coli infection of humans and animal causes worse of the disease thus not recommended; despite this, where is the drug resistance come from?

**Authors response:** It is a good comment. In most laboratory setups of the country, the specific bacterial organism that causes diarrhea has not been identified rather is reported as bacteria if there are pus cells in the field. Based on this simple report, the prescriber just prescribes any of the antibiotics that are available in health institutions. Therefore; the patient may be repeatedly exposed to these antibiotics and due to lack of knowledge, most patients did not have the habit of taking medicine completely which altogether contributes to the development of resistance. Another possibility is that mutant bacteria may be directly reached to the patient and consequently cattle can consume these mutant bacteria or vice versa.

9. **Comment**: In addition, u tested the number of antibiotics that are ban and not used in animals? E.g., Chloramphenicol; what is the reason for the discrepancy**?**

**Authors response:** Thank you! for the comment. Even though sensitivity tests had been done for banned antibiotics in cattle, it still gives a clue that the cattle can consume the already resistant bacteria for the drug. It also gives information on the transmission of STEC O157:H7 from humans, the environment, plants, soil, and water to animals.

We thank all the reviewers for giving comments and supportive suggestions in order to improve the paper**!**

Please note all the corrections made were labeled in red color in the main text.

With best regards

Tigist Engda (PI and corresponding author)
