# Supplementary material for: Shiga toxin-producing Escherichia coli O157:H7 among diarrheic patients and their cattle in Amhara National Regional State, Ethiopia
Source: PLoS One. 2023 Dec 21;18(12):e0295266. doi: 10.1371/journal.pone.0295266 (PMC10734908; doi:10.1371/journal.pone.0295266)
Supplement: Attachment — Submitted filename: Response to academic Editors-R3.docx [file pone.0295266.s005.docx]

Date: October 29, 2023

**To:** PLOS ONE

Editor-In-Chief

**From:** Tigist Engda (PI and corresponding author)

**Submission ID**: PONE-D-23-13678

**Title:** Shiga toxin-producing Escherichia coli O157:H7 among diarrheic patients and their cattle in Amhara National Regional State, Ethiopia

**Point by point authors’ response**

First, we would like to thank and appreciate the reviewers for their critical and constructive comments. We attempted all the questions and concerns raised by academic editors.

**ACADEMIC EDITOR’S COMMENTS:**

1. **Comment**: Thank you for stating the following financial disclosure:

[ The Funder has no any role in the study design, data collection and analysis, decision to publish, or preparation of the manuscript]

At this time, please address the following queries:

a. **Comment**: Please clarify the sources of funding (financial or material support) for your study. List the grants or organizations that supported your study, including funding received from your institution.

**Author response:** Thank you, there is no funding for this study.

b. **Comment**: State what role the funders took in the study. If the funders had no role in your study, please state: “The funders had no role in study design, data collection and analysis, decision to publish, or preparation of the manuscript.”

**Author response:** Thank you. Since there is no specific fund for the study, no funders took any role in the study.

c. **Comment**: If any authors received a salary from any of your funders, please state which authors and which funders.

**Author response:** Thank you. We all are academic staffs of the University and no one from the authors received any salary for being a member of this study.

d. **Comment:** If you did not receive any funding for this study, please state: “The authors received no specific funding for this work.”

**Author response:** Thank you! We had been stated as “no specific grant” in funding subtitle of the main document and now we are corrected it as ‘‘The authors received no specific funding for this work.’’ page 30.

- Please include your amended statements within your cover letter; we will change the online submission form on your behalf.

**Author response:** Thank you!

- We would like to thank PLOS ONE academic editors and reviewers for giving their precious time to see the document thoroughly, and raised very helpful comments and suggestions in order to improve the paper**!**

We look forward your response!

With best regards!

Tigist Engda (PI and corresponding author)
