# Supplementary material for: Shiga toxin-producing Escherichia coli O157:H7 among diarrheic patients and their cattle in Amhara National Regional State, Ethiopia
Source: PLoS One. 2023 Dec 21;18(12):e0295266. doi: 10.1371/journal.pone.0295266 (PMC10734908; doi:10.1371/journal.pone.0295266)
Supplement: Attachment — Submitted filename: Response to Reviewers-R4.docx [file pone.0295266.s007.docx]

Date: November 12, 2023

**To:** Plose one

Editor-In-Chief

**From:** Tigist Engda (PI and corresponding author)

**Submission ID**: PONE-D-23-13678 R2

**Title:** Shiga toxin-producing Escherichia coli O157:H7 among diarrheic patients and their cattle in Amhara National Regional State, Ethiopia

**Point by point authors’ response**

First, we would like to thank and appreciate the reviewers for their critical and constructive comments. We attempted all the questions and concerns raised by academic editors.

**ACADEMIC EDITOR’S COMMENTS:**

1. **Requirement at time of submitting revised manuscript:**

Please include the following items when submitting your revised manuscript:

•	A rebuttal letter that responds to each point raised by the academic editor and reviewer(s). You should upload this letter as a separate file labeled 'Response to Reviewers'.

•	A marked-up copy of your manuscript that highlights changes made to the original version. You should upload this as a separate file labeled 'Revised Manuscript with Track Changes'.

•	An unmarked version of your revised paper without tracked changes. You should upload this as a separate file labeled 'Manuscript'.

**Author response:** Thank you! We have included all items; the rebuttal letter (Response to Reviewers), a marked-up copy of the manuscript with file name “Revised Manuscript -R4 with Track changes” and unmarked version of the original file with file name “Manuscript” are attached and you are kindly requested to upload these files.

**II. Comment:** Please insert comments here and delete this placeholder text when finished.

Be sure to:

1. Indicate which changes you require for acceptance versus which changes you recommend

**Authors response:** Thank you. We consider it.

2. Thank you for addressing all comments and suggestions in the previous version. Here, I attached some minor comments as track changes in the main manuscript.

**Author response:** Thank you. We have noted and corrected it point by point in the manuscript and manuscript-R4 with track changes.

2.1. **Comment:** Line 48: the word **‘**continues’ change by ‘continued’

**Author response:** Thank you for the comment and corrected it on page3, line 48.

2.2. **Comment:** Line 173: The word ‘which’ change by ‘that’

**Author response:** Thank you. We have noted and corrected it on page 8, line 171.

2.3. **Comment:** Line 206: The word ‘encoded’ change by ‘encoding’

**Author response:** Thank you. We have noted and corrected it on page 10, line 204.

2.4**. Comment:** Line 223**:** add the letter ‘a’ before the word gel.

**Author response:** Thank you for the comment and corrected it on page 10, line 220.

2.5. **Comment:** Line 243: Delete the word ‘by’.

**Author response:** Thank you for the comment and corrected it on page11, line 238.

2.6. **Comment:** Line 262: The word ‘prior to’ change by ‘before’ and add the word ‘the’ before training.

**Author response:** Thank you. We have noted and corrected it on page 12, line 257.

2.7**. Comment:** Line274-276: sentences state in this line and the sentences on line 273-274 are opposites; please remove this one. You only variables with p<0.2 in bivariate to multivariable regression.

**Author response:** Thank you. We accepted the comment and corrected it on page 13. Line 267-270.

2.8. **Comment:** Line 281: edit the word ‘lemeshow’ by ‘Lemeshow’

**Author response:** Thank you for the comment and corrected it on page 13, line 274.

2.9. **Comment:** Line 290: Delete the word on

**Author response:** Thank you for the comment and corrected it on page13, line 282.

2.10**. Comment:** Line 293: Delete H7

**Author response:** Thank you for the comment. We were communicated with those who were STEC O157:H7 positive patients not all STEC O157 positive patients. Basically, STEC O157:H7 is one group of the STEC O157. So, it should be stated as STEC O157:H7. Because our aim is to find out only STEC O157:H7.

2.11**. Comment**: Line 319: The word ‘from’ change by the word ‘of’

**Author response**: Thank you. we are corrected on page 16, line 313.

2.12**. Comment**: Line 322: the word ‘swabs’ change to ‘swab’

**Author response**: Thank you. we are corrected on page16, line 315.

2.13**. Comment:** Line 334 and 335: the word ‘were’ and resident change by ‘was’ and ‘residents’ respectively

**Author response:** Thank you. We are corrected on page 18. Line 327 and 328 respectively.

2.14. **Comment:** Line 347, table 4: What is the value of AOR for drinking water source and drinking water treatment?

**Author response:** Thank you. We are now incorporated the AOR value of drinking water source and drinking water treatment on page 20, line 340 in table 4.

2.15**. Comment:** Line 347, table 4: why do you include this variable in Multivariable regression because the p value is greater than your selection criteria, P<0.2 in History of hand washing habit independent variable.

**Author response:** Thank you for the comment. The P-value of after eating, one of the categories under History of hand washing habit variable, is (0.00) which is less than 0.2. and needs to calculate Multivariable regression to know it’s association. Similarly drinking water treatment variable too.

2. 16**. Comment:** Line 377: change the word ‘which’ by ‘that’.

**Author response:** Thank you. We are corrected on page 24, line 369.

2.17**. Comment:** Line 380: change the word ‘antibiotics’ to ‘antibiotic’.

**Author response:** Thank you and corrected on page 24, line 372

2.18**. Comment:** Line 393: delete the word ‘and” before the highest.

**Author response:** Thank you and corrected on page 26, line 385.

2.19**. Comment:** Line 393: add hyphen b/n under and five**.**

**Author response:** Thank you and corrected on page 26, line 385.

2.20. **Comment:** line 394: add the word ‘of’ after 11.7% and after 6.1%.

**Author response:** Thank you and corrected on page 26, line 386.

2.21**. Comment:** Line 396: change the word ‘were’ by ‘was.

**Author response:** Thank you and corrected on page 26, line 388.

2.22**. Comment**: Line 407: Change the word ‘traditionally’ to ‘traditional’.

**Author response:** Thank you and corrected on page 26, line 398.

2.23. **Comment:** Line 413: change the word ‘to’ by ‘in’.

**Author response:** Thank you and corrected on page 26, line 404.

2.24**. Comment:** line 414: add hyphen after the word ‘under’, change the word ‘was’ by ‘were’ and ‘to’ by ‘for’.

**Author response:** Thank you and corrected on page 27, line 405.

2.25**. Comment:** line 415 and 416: add ‘s’ to the word system and practice and the before time.

**Author response:** thank you and corrected on page 27, line 406.

**2.26. Comment**: Line 419: the word had been changed by were.

**Author response**: Thank you and corrected on page 27, line 409.

2.27**. Comment:** Line 421: change the word ‘are’ by ‘is’.

**Author response:** Thank you and corrected on page 27, line 412.

2.28**. Comment:** Line 426: change the word was by were.

**Author response:** Thank you and corrected on page 27, line 417.

2.29**. Comment:** Line 434: add the word ‘and’ before Mexico and Line 435: add the word ‘and’ before the type of samples**.**

**Author response:** Thank you and corrected on page 28, and line 425 and 426.

2.30. **Comment:** Line 451: Delete the word ‘in’ before different.

**Author response**:  Thank you and corrected on page 28, line 442.

2.31**. Comment:** Line 460: add word ‘and’ before means, ‘comma’ after transmission and ‘a’ before prevention.

**Author response:** Thank you. we have incorporated the word “and” before the word “means” added comma after transmission and “a” before prevention. Page 29, line 451.

2.32. **Comment:** Line 473: add ‘ing’ to the word ‘teach’

**Author response:** Thank you and corrected on page 29, line 463.

2.33**. Comment:** Line 475: add the word ‘and’ before the word after

**Author response:** Thank you and corrected on page 29, line 465

2.34. **Comment:** Line477: change the word ‘with respect to’ by ‘concerning’.

**Author response**: Thank you and changed the word on page 29, line 466.

NB: We appreciate the academic editors for critically checked the manuscript and giving us valuable comments and of course we have tried to attempt all the comments.

With best regards!

Tigist Engda (PI and corresponding author)
